# Design, Synthesis, and Integrated In Silico and In Vitro Evaluation of Chloro-Substituted Salicylaldehyde Benzoylhydrazones as Anticancer Agents

**DOI:** 10.3390/ph19091387

**Published:** 2026-09-01

**Authors:** Boryana Nikolova-Mladenova, Rositsa Mihaylova, Stilyana Kostova, Boris Vasilev, Irini Doytchinova, Mariyana Atanasova

**Affiliations:** 1 Department of Chemistry, Faculty of Pharmacy, Medical University of Sofia, 2 Dunav Str., 1000 Sofia, Bulgaria; 120588@pharmfac.mu-sofia.bg (B.V.); idoytchinova@pharmfac.mu-sofia.bg (I.D.); 2 Centre of Excellence in Informatics and Information and Communication Technologies, 1113 Sofia, Bulgaria; rmihaylova@pharmfac.mu-sofia.bg; 3 Department of Pharmacology, Pharmacotherapy and Toxicology, Faculty of Pharmacy, Medical University of Sofia, 2 Dunav Str., 1000 Sofia, Bulgaria; 105345@students.mu-sofia.bg

**Keywords:** chloro-salicylaldehyde, hydrazones, antileukemic activity, selectivity, BCR–ABL1 fusion protein

## Abstract

**Background**: Building on earlier work with methoxy-, dimethoxy-, bromo-, and nitro-substituted salicylaldehyde benzoylhydrazones, we synthesized and evaluated new 4-chloro- and 5-chloro derivatives bearing an additional chlorine substituent on the hydrazide-derived phenyl ring to further explore the impact of halogenation on cytotoxic activity. **Methods**: Chloro-hydrazones were obtained through a one-step condensation of 4- or 5-chlorosalicylaldehyde with benzhydrazide or 2-, 3-, and 4-chlorobenzhydrazides. Their physicochemical, pharmacokinetic, ADME, lead-likeness, and drug-likeness profiles were evaluated in silico using SwissADME, ACD/Labs v9.10, and MDL QSAR v2.2.0.0.446. The synthesized compounds were structurally characterized by IR, ^1^H NMR, ^13^C NMR, and HR ESI–MS, and their cytotoxic activity was subsequently assessed by the MTT assay in selected cancer cell lines. Molecular docking was performed against ABL1 tyrosine kinase (ABL1 TK), a potential molecular target relevant to two of the investigated leukemia cell lines, using GOLD v.5.2.2 (CCDC Ltd., Cambridge, UK). **Results**: The chloro-substituted salicylaldehyde benzoylhydrazones exhibited pronounced, cell-type-dependent cytotoxicity, with leukemia cells being substantially more sensitive than breast carcinoma cells. Most derivatives showed low- to sub-micromolar IC_50_ values against SKW-3 human T-cell prolymphocytic leukemia, K-562 human chronic myeloid (myelogenous) leukemia, and BV-173 human BCR::ABL1-positive leukemia cells, with **K3** and **K4** exhibiting the highest activity in SKW-3 cells (IC_50_ = 0.5 ± 0.1 µM). **K5** showed particularly strong activity against K-562 and BV-173 cells (IC_50_ = 0.7 ± 0.1 and 0.9 ± 0.1 µM, respectively), compared with 26.9 ± 2.4 and 21.5 ± 3.3 µM for imatinib. HL-60 human acute promyelocytic leukemia cells showed intermediate sensitivity (IC_50_ = 1.3–13.9 µM), whereas the activity against MCF-7 estrogen receptor-positive and MDA-MB-231 triple-negative breast carcinoma cells was more variable (1.5–32.1 and 4.1–62.5 µM, respectively). The derivatives showed high selectivity toward malignant cells relative to non-malignant CCL-1 non-malignant mouse fibroblasts, with lower-bound SI values frequently exceeding 50 and reaching >200 in SKW-3 cells. **Conclusions**: The chloro-substituted salicylaldehyde benzoylhydrazones exhibited pronounced and selective cytotoxicity, particularly toward leukemia cell lines, identifying this scaffold as a promising starting point for further anticancer drug-discovery studies.

## 1. Introduction

Hydrazone chemistry has emerged as a versatile platform in medicinal chemistry, owing to the structural adaptability of the hydrazone motif and its capacity to generate diverse molecular architectures with tunable physicochemical and biological properties [1,2,3,4]. The combination of imine and hydrazide functionalities within this motif confers versatile coordination behavior, tautomeric flexibility, and the capacity to engage in diverse interactions with biological targets [3,5,6]. Accordingly, hydrazone derivatives have been investigated for a wide range of biological activities, including antimicrobial, antifungal, antiviral, antitubercular, anti-inflammatory, antioxidant, anticonvulsant, antiparasitic, and, particularly, anticancer effects [7,8,9,10,11,12,13,14,15,16,17,18,19,20,21,22,23,24]. Several hydrazone-containing compounds have also progressed into clinical applications or serve as valuable pharmacophores in drug discovery, highlighting the pharmaceutical relevance of this scaffold [25].

Among hydrazones, salicylaldehyde-based derivatives are of particular interest because the ortho-hydroxyl group adjacent to the azomethine bond enables keto-enol tautomerism and intramolecular hydrogen bonding, thereby influencing molecular geometry, electronic distribution, stability, and biological activity [26,27]. The presence of donor oxygen and nitrogen atoms additionally facilitates metal coordination and diverse intermolecular interactions. Importantly, the biological properties of salicylaldehyde benzoylhydrazones can be modulated through substitution of either the aldehyde-derived or hydrazide-derived aromatic ring. Electron-donating and electron-withdrawing substituents can alter electron density, hydrogen-bonding properties, lipophilicity, and molecular conformation, thereby affecting biological activity. Consequently, salicylaldehyde benzoylhydrazones and their substituted analogs have been extensively investigated for cytotoxic, antibacterial, antioxidant, anti-inflammatory, and enzyme inhibitory properties [10,28,29,30], with their anticancer activity being of particular interest.

Over recent years, we have systematically investigated the cytotoxic potential of salicylaldehyde-based benzoylhydrazones [31,32,33,34,35,36,37]. Our previous studies demonstrated that relatively subtle modifications of the salicylaldehyde and hydrazide fragments can substantially influence cytotoxic potency and selectivity. In particular, methoxy-substitution proved highly effective: derivatives obtained from 3-methoxysalicylaldehyde showed strong cytotoxicity toward AML HL-60 cells [31,32], whereas 5-methoxy-substituted analogs exhibited potent activity against MCF-7 breast cancer cells and favorable selectivity toward malignant cells [33]. Nitro- and bromo-substituted derivatives also showed pronounced activity against leukemic cell lines, including HL-60, SKW-3, and BV-173 [34,35]. More recently, 4-methoxysalicylaldehyde-based hydrazones demonstrated remarkable anticancer activity at nanomolar concentrations against leukemia HL-60 and K-562 cell lines and breast MCF-7 cancer cells, together with high selectivity and specificity [36]. Furthermore, selected dimethoxy-substituted derivatives displayed pronounced selectivity toward leukemic cells showing limited effects on normal HEK 293 cells [37]. Collectively, these findings demonstrate that relatively small electronic and steric modifications within the salicylaldehyde and hydrazide fragments can markedly influence the cytotoxic profile of this scaffold.

These observations prompted us to investigate additional substituents capable of modulating the physicochemical and biological properties of salicylaldehyde benzoylhydrazones. Among the substituents widely employed in medicinal chemistry, halogens, particularly chlorine, are of considerable interest [38,39,40]. Chlorine is frequently incorporated into pharmaceutical compounds because it can substantially influence potency, pharamacokinetic parameters, including clearance, half-life, and drug exposure in vivo, binding affinity, selectivity, and cell permeability [38,41]. Approximately one-quarter of approved drugs and nearly 40% of lead compounds contain halogens [38,42], while a substantial proportion of recently FDA-approved small-molecule drugs also contain chlorine [42]. Chlorine substitution may increase lipophilicity and membrane permeability, reduce susceptibility to oxidative metabolism, and strengthen ligand–target interactions through hydrophobic interactions and halogen bonding effects [38,41]. At the same time, its inductive electron-withdrawing effect and ability to participate in resonance interactions can modify electronic properties and reactivity of aromatic systems.

Importantly, the position of chlorine substitution may be as relevant as its presence. Positional isomers can exhibit substantial differences in steric effects, electronic distribution, molecular dipole moment, conformational preferences, intermolecular interactions, lipophilicity, solubility, and protein binding despite having identical elemental composition. Salicylaldehyde hydrazones provide a suitable framework for systematically investigating such effects because chlorine can be introduced independently into both the aldehyde-derived and hydrazide-derived aromatic rings. Simultaneous variation in these substitution positions allows modification of molecular properties while maintaining the same central hydrazone pharmacophore. Because the biological consequences of chlorine positioning arise from the combined effects of electronic, steric, conformational, and intermolecular factors, they cannot be reliably predicted from general substituent effects alone. Systematic investigation of well-defined positional isomeric series is therefore needed to establish meaningful structure–property and structure–activity relationships.

In the present study, a series of eight chloro-substituted salicylaldehyde benzoylhydrazones was designed and synthesized by condensation reactions of 4- or 5-chlorosalicylaldehyde with benzhydrazide and 2-, 3-, or 4-chlorobenzhydrazides. This design generated a structurally homogeneous series in which chlorine substitution was varied on the aldehyde-derived and hydrazide-derived aromatic rings. The synthesized compounds were structurally characterized, and their molecular descriptors, drug-likeness, and ADME-related properties were evaluated using contemporary in silico approaches. Their in vitro cytotoxic activities were also determined to investigate relationships between chlorine substitution pattern, predicted pharmacokinetic properties, and biological activity. The combined experimental and computational investigation provides new insight into the structure–property and structure–activity relationships of chloro-substituted salicylaldehyde benzoylhydrazones and may contribute to the rational design of improved hydrazone-based bioactive molecules.

## 2. Results

### 2.1. Design of Chloro-Substituted Salicylaldehyde Benzoylhydrazones and In Silico Drug Likeness Evaluation

In our earlier investigations, we systematically modified salicylaldehyde benzoylhydrazones by introducing a range of substituents—such as bromo atoms, nitro groups, and methoxy groups—at different positions on the aromatic framework. These structural variations were subsequently subjected to comprehensive in silico analyses alongside experimental biological evaluation against a panel of anticancer cell lines [31,32,33,34,35,36,37]. The outcomes of these studies provided valuable understandings into the structure–activity relationships governing their anticancer potential.

Based on these results, we directed our attention toward the design and exploration of chloro-substituted analogs. Specifically, we selected 4-chloro and 5-chloro salicylaldehyde derivatives as promising candidates for further investigation (Table 1). In the next phase, chloro substituents were strategically incorporated not only into the phenyl ring of the salicylaldehyde core but also into the phenyl ring of the hydrazide moiety. This dual modification approach was intended to assess the influence of chloro substitution on the physicochemical properties, molecular interactions, and potential biological activity of the resulting compounds.

As an initial step prior to the synthesis of the target molecules, an extensive in silico analysis was performed to evaluate the molecular characteristics of the chloro-substituted hydrazones according to multiple relevant criteria. This analysis included an assessment of drug-likeness, key physicochemical parameters, and ADME (absorption, distribution, metabolism, and excretion) properties. Specifically, the evaluated descriptors included molecular weight, predicted pKa, proportion of ionized species, lipophilicity (logP), polar surface area, number of rotatable bonds, hydrogen bond donors and acceptors, as well as any deviations from Lipinski’s rule of five. In addition, aqueous solubility, gastrointestinal absorption, oral bioavailability, bioavailability score, blood–brain barrier permeability, potential inhibition of cytochrome P450 isoenzymes, likelihood of interaction with P-glycoprotein, lead-likeness, and synthetic accessibility were also examined (Table 2). These predictions were generated using the SwissADME online platform (https://www.swissadme.ch/, accessed on 9 January 2026) in combination with ACD/Labs Suite v. 9.10 (Advanced Chemistry Development, Inc., Toronto, ON, Canada).

Moreover, key pharmacokinetic parameters were estimated using previously developed quantitative structure–pharmacokinetic relationship (QSPkR) models [43,44,45]. The predicted parameters included the fraction of unbound drug in plasma, total systemic clearance, and volume of distribution at steady state (Table 2).

The molecular weights (Mws) of the compounds, spanning from 274.7 to 309.15 g/mol, suggest that the novel chloro-substituted hydrazones are more appropriately classified as lead-like rather than drug-like [46]. Their relatively low molecular weight leaves room for further structural modification and optimization aimed at improving anticancer activity and selectivity. The compounds behave as weak acids, with predicted pK_a_ values between 7.22 and 7.87, showing a clear dependence on substitution pattern. Specifically, 4-chloro-salicylaldehyde derivatives exhibit higher acidity than their 5-chloro counterparts. At physiological pH (7.4), the estimated fraction of ionized species (f_A_) is approximately 53–60% for 4-chloro derivatives and 25–30% for 5-chloro derivatives.

The calculated consensus logP values for the designed series of chloro-substituted salicylaldehyde benzoylhydrazones (**K1**–**K8**) fall within a relatively narrow range (2.93–3.48), indicating moderate lipophilicity across all compounds. This range is typically seen as optimal for drug-like compounds because it strikes a balance between adequate membrane permeability and reasonable water solubility. A clear trend is observed when comparing mono- and di-chloro-substituted derivatives. The parent compounds **K1** and **K5**, which contain only a single chloro substituent on the salicylaldehyde moiety, exhibit the lowest lipophilicity (logP ≈ 2.93–2.95). Introduction of an additional chloro atom into the hydrazide phenyl ring (**K2**–**K4** and **K6**–**K8**) consistently increases logP values to approximately 3.40–3.48. This effect can be attributed to the electron-withdrawing and hydrophobic nature of the chloro substituent, which enhances the overall nonpolar character of the molecule. The position of the chloro substituent on the hydrazide ring exerts a light influence on lipophilicity. Among the 4-chlorosalicylaldehyde derivatives (**K1**–**K4**), the meta-substituted analog **K3** (3-chloro) displays the highest logP (3.48), followed closely by the ortho (**K2**, 3.46) and para (**K4**, 3.41) derivatives. A similar trend is observed in the 5-chlorosalicylaldehyde series (**K5**–**K8**), although the differences are less pronounced.

A polar surface area (PSA) value of 61.69 Å^2^ is consistent with drug-likeness and absorption criteria (PSA ≤ 140 Å^2^), suggesting favorable cell membrane permeability and high intestinal absorption (>90%) [47,48,49,50]. Moreover, this PSA value falls within the range commonly associated with central nervous system (CNS)-active compounds (PSA < 60–90 Å^2^), indicating a potential for blood–brain barrier (BBB) penetration [51,52]. Although each molecule contains four freely rotatable bonds, a relatively rigid conformation is maintained due to p–π conjugation between the aromatic rings and the hydrazone moiety. With two hydrogen bond donors and three hydrogen bond acceptors, together with favorable molecular weight values, all compounds comply with Lipinski’s rule of five. A slight exceedance of the recommended LogP threshold is observed for four derivatives, namely the 3- and 4-chloro-substituted benzoylhydrazones (**K3**, **K4**, **K7**, and **K8**).

Regarding ADME properties, the chloro-substituted derivatives exhibit moderate aqueous solubility and high predicted gastrointestinal (GI) absorption. They show an estimated 55% probability of achieving oral bioavailability above 10% in rats (BA score), which is associated with their low fraction of Csp^3^ atoms—a descriptor commonly linked to favorable oral performance prior to advanced experimental evaluation. In addition, the compounds are predicted to be permeable across the BBB. All derivatives are predicted to inhibit CYP1A2, CYP2C19, and CYP2C9, with the exception that **K1** and **K5** are not predicted inhibitors of CYP2C19. None of the compounds are predicted to be substrates of P-glycoprotein. Overall, the molecules display favorable drug-likeness profiles; however, their predicted XLOGP values exceed 3.5, placing them outside the typical lead-likeness space (250 ≤ Mw ≤ 350 g/mol, XLOGP ≤ 3.5, and FRB ≤ 7) [53]. Synthetic accessibility scores ranging from 2.32 to 2.47 further indicate that these compounds should be relatively easy to synthesize.

Based on the predicted pharmacokinetic parameters, all compounds exhibit small steady-state distribution volumes (VDss; 0.204–0.218 L/kg), and the values are close to the volume of extravascular body water [43,44,45]. The very low fraction of unbound drug in plasma (f_u_ = 0.017–0.057) indicates extensive plasma protein binding, estimated at approximately 94–98, which is consistent with the known tendency of acidic drugs to bind strongly to human serum albumin [54]. Large-scale analyses of drugs across different ionization states indicate that the majority of anionic compounds (78%) are characterized by low systemic clearance (CL < 0.24 L/h/kg), while only a small fraction (1–2%) exhibit high clearance values (CL > 0.96 L/h/kg) [55]. In contrast, the predicted total clearance values of the novel compounds range from 0.66 to 1.59 L/h/kg, classifying most of them as high-clearance compounds (CL ≥ 0.96 L/h/kg). Only two compounds, **K6** and **K7**, fall into the moderate clearance category, with CL values of 0.66 and 0.92 L/h/kg, respectively.

Overall, the in silico drug-likeness screening of the designed chloro-substituted salicylaldehyde benzoylhydrazones indicates promising properties, supporting their selection for synthesis and further experimental evaluation.

### 2.2. Synthesis of Chloro-Substituted Salicylaldehyde Benzoylhydrazones

**Figure 1:**
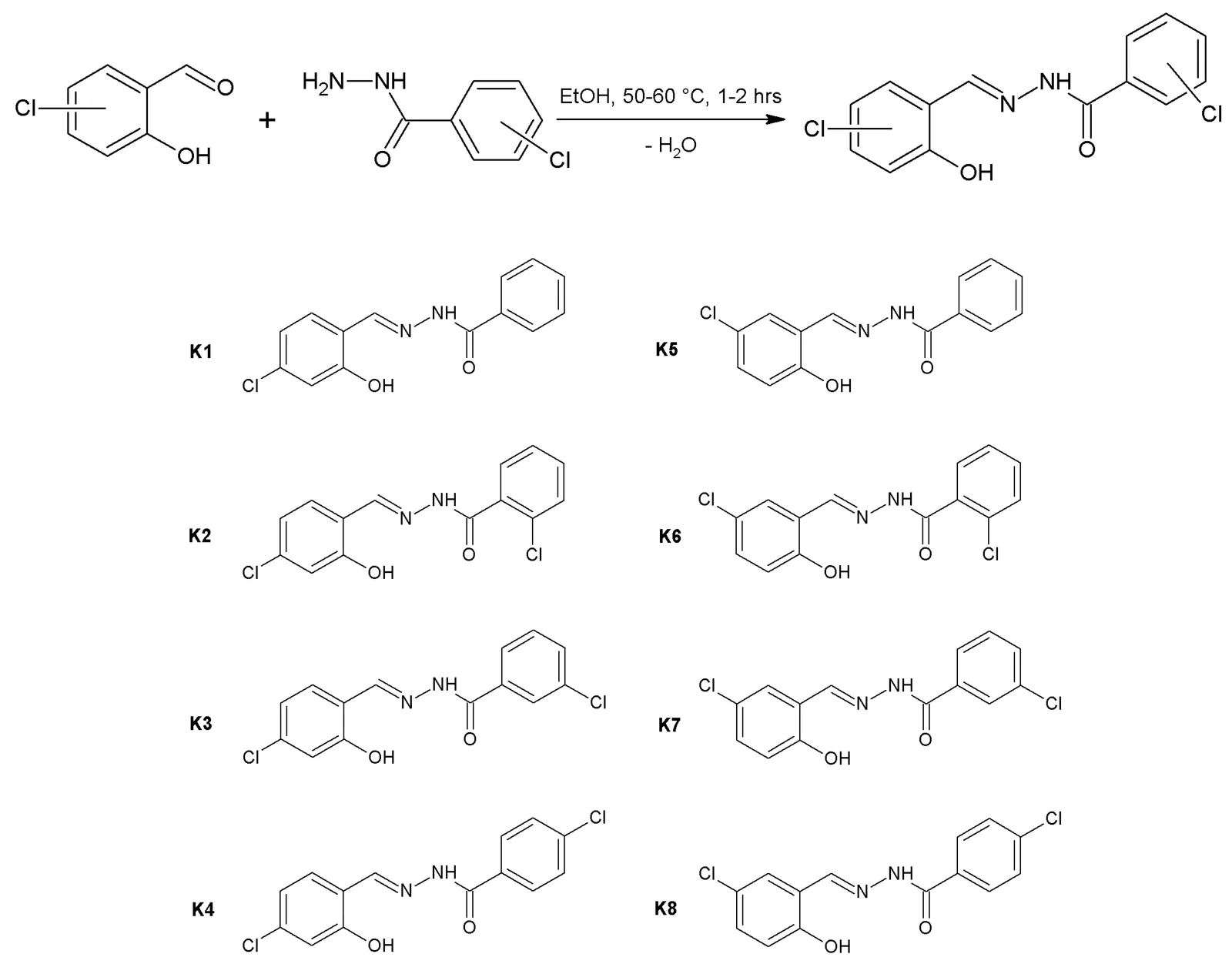
Scheme of synthesis and structures of chloro-substituted salicylaldehyde benzoylhydrazones (**K1**–**K8**).

The chloro-substituted salicylaldehyde benzoylhydrazones were obtained by a one-step condensation reaction of 4- or 5-chlorosalicylaldehyde with benzhydrazide or the corresponding 2-, 3-, or 4-chloro-substituted benzhydrazides in ethanol (Figure 1) [31,32,33,34,35,36,37]. The synthesized derivatives were isolated as crystalline solids in good yields.

Their molecular compositions were established by elemental analysis and high-resolution electrospray ionization mass spectrometry (HR ESI–MS). The HR ESI–MS measurements, recorded in positive-ion mode, showed the expected protonated molecular ions [M + H]^+^ together with the characteristic chlorine isotope patterns. In particular, the presence of one or two chlorine atoms resulted in isotope peaks separated by 2 Da, reflecting the natural abundance of ^35^Cl and ^37^Cl. Further structural characterization was achieved by IR, ^1^H NMR, and ^13^C NMR spectroscopy, with the complete spectroscopic data provided in Supporting Information.

The IR spectra provided evidence for formation of the hydrazone linkage, with the C=N stretching vibration appearing in the range of 1593–1606 cm^−1^. Absorptions attributable to the phenolic OH and hydrazone NH groups were observed at 3202–3418 and 3148–3216 cm^−1^, respectively. The strong C=O absorption bands at 1635–1653 cm^−1^ are consistent with the keto form of the hydrazones in the solid state.

The NMR data further supported the proposed structures. In the ^1^H NMR spectra, the characteristic azomethine proton (HC=N) resonated at 8.48–8.64 ppm, whereas the phenolic OH signal appeared as a broad singlet at 12.15–12.25 ppm. The NH protons were detected in the 11.04–11.59 ppm range. The ^13^C NMR spectra showed the expected resonances for the azomethine and carbonyl carbons at 146.11–147.25 and 161.93–163.45 ppm, respectively.

### 2.3. In Vitro Cytotoxic Activity and Selectivity

**Figure 2:**
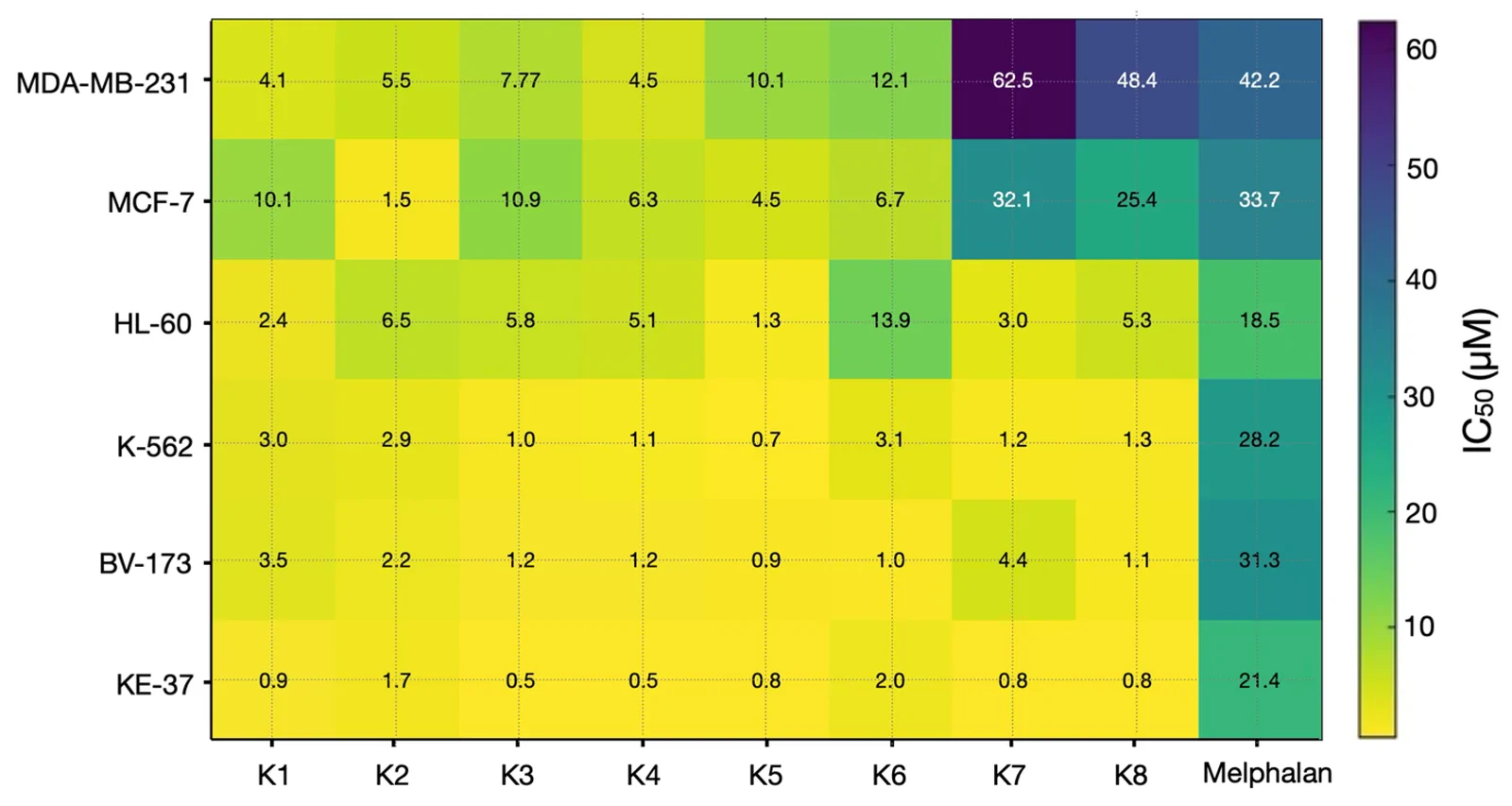
IC_50_ heatmap, representing the cytotoxic potency of the chlorosalicylaldehyde benzoylhydrazone derivatives (**K1**–**K8**) versus melphalan in breast carcinoma and leukemia cell lines.

The cytotoxic potential of eight newly synthesized chlorosalicylaldehyde benzoylhydrazone derivatives (**K1**–**K8**) was evaluated against epithelial breast cancer cell lines (MDA-MB-231, MCF-7), human leukemic cell lines (SKW-3, HL-60, K-562, and BV-173), and the non-malignant fibroblast line CCL-1. Melphalan, an established alkylating agent in clinical use, was included as a reference compound. Imatinib was included as reference compound specifically for the BCR::ABL1-positive K-562 and BV-173 cell lines. IC_50_ values were determined for all cell lines (Table 3, Figure 2).

A consistent trend was observed in which leukemic cell lines were considerably more sensitive to the hydrazone derivatives than epithelial breast cancer lines. Across the leukemic panel, IC_50_ values were generally in the low micromolar or sub-micromolar range, substantially exceeding the potency of the reference drug melphalan. SKW-3 cells, derived from T-cell prolymphocytic leukemia, were exceptionally sensitive, with most compounds exhibiting sub-micromolar IC_50_ values (0.5–0.9 µM), with the exceptions of **K2** (1.7 µM) and **K6** (2.0 µM). Compounds **K3** and **K4** were particularly potent, displaying IC_50_ values as low as 0.5 µM, representing more than a 40-fold increase in activity compared with melphalan (21.4 µM). Comparable potency was observed in chronic myeloid leukemia (CML)-derived K-562 and BV-173 cells. Compounds **K3**, **K4**, and **K5** achieved IC_50_ values near or below 1 µM in both cell lines, while **K7** (1.2 µM) and **K8** (1.3 µM) were similarly active in K-562 cells, and **K6** (1.1 µM) and **K8** (1.1 µM) in BV-173 cells; all demonstrated markedly greater potency than melphalan (28.2 and 31.3 µM, respectively). In contrast, the promyeloblast HL-60 leukemic cell line exhibited intermediate and variable chemosensitivity, allowing classification into the following three relative activity ranges within this cell line: the most active compounds, with IC_50_ values of 1.3–3 µM (**K1**, **K5**, and **K7**); moderately active compounds, with IC_50_ values between 5.1 and 6.5 µM (**K2**, **K3**, **K4**, and **K8**); and one less active compound, **K6**, with an IC_50_ value of 13.9 µM, comparable to the reference melphalan (18.5 µM). These lineage-dependent differences in sensitivity may reflect distinct molecular characteristics of the respective leukemia models. In particular, the pronounced sensitivity of the BCR::ABL1-positive K-562 and BV-173 cells may indicate a potential relationship with BCR::ABL1-associated molecular features, whereas the comparatively lower sensitivity of HL-60 cells could potentially involve differences in drug-handling mechanisms, including efflux activity. However, these interpretations remain hypotheses and require further experimental validation.

By contrast, epithelial breast carcinoma lines exhibited a broader and more heterogeneous response. The triple-negative MDA-MB-231 line was least sensitive; within this line, compounds could be grouped into the following three activity ranges: active compounds (**K1**, **K2**, and **K4**) with IC_50_ values of approximately 4–5 µM, corresponding to roughly ten-fold greater potency than melphalan (42.2 µM); moderately active compounds (**K3**, **K5**, and **K6**) with IC_50_ values between 7.7 and 12.1 µM; and weakly active compounds (**K7** and **K8**), which required concentrations exceeding 40 µM to achieve 50% growth inhibition and showed activity comparable to or lower than that of the reference drug. Despite the chemoresistant phenotype characteristic of triple-negative MDA-MB-231 cells, compounds **K1**, **K2**, and **K4** retained appreciable activity. In contrast, MCF-7 cells, representing an estrogen receptor-positive luminal subtype, were more responsive overall. Compound **K2** (IC_50_ = 1.5 µM) was particularly effective, exhibiting more than a 22-fold increase in potency compared with melphalan (33.7 µM). Compounds **K5** (4.5 µM), **K4** (6.3 µM), and **K6** (6.7 µM) also demonstrated approximately an order of magnitude greater activity than melphalan, whereas **K1** (10.1 µM) and **K3** (10.9 µM) were only about three-fold more potent. Compounds **K7** and **K8** showed activity comparable to that of melphalan. The observed differences in susceptibility between MDA-MB-231 and MCF-7 cells are consistent with clinical observations that estrogen receptor-positive breast cancers are generally more chemosensitive than the triple-negative phenotype.

**Figure 3:**
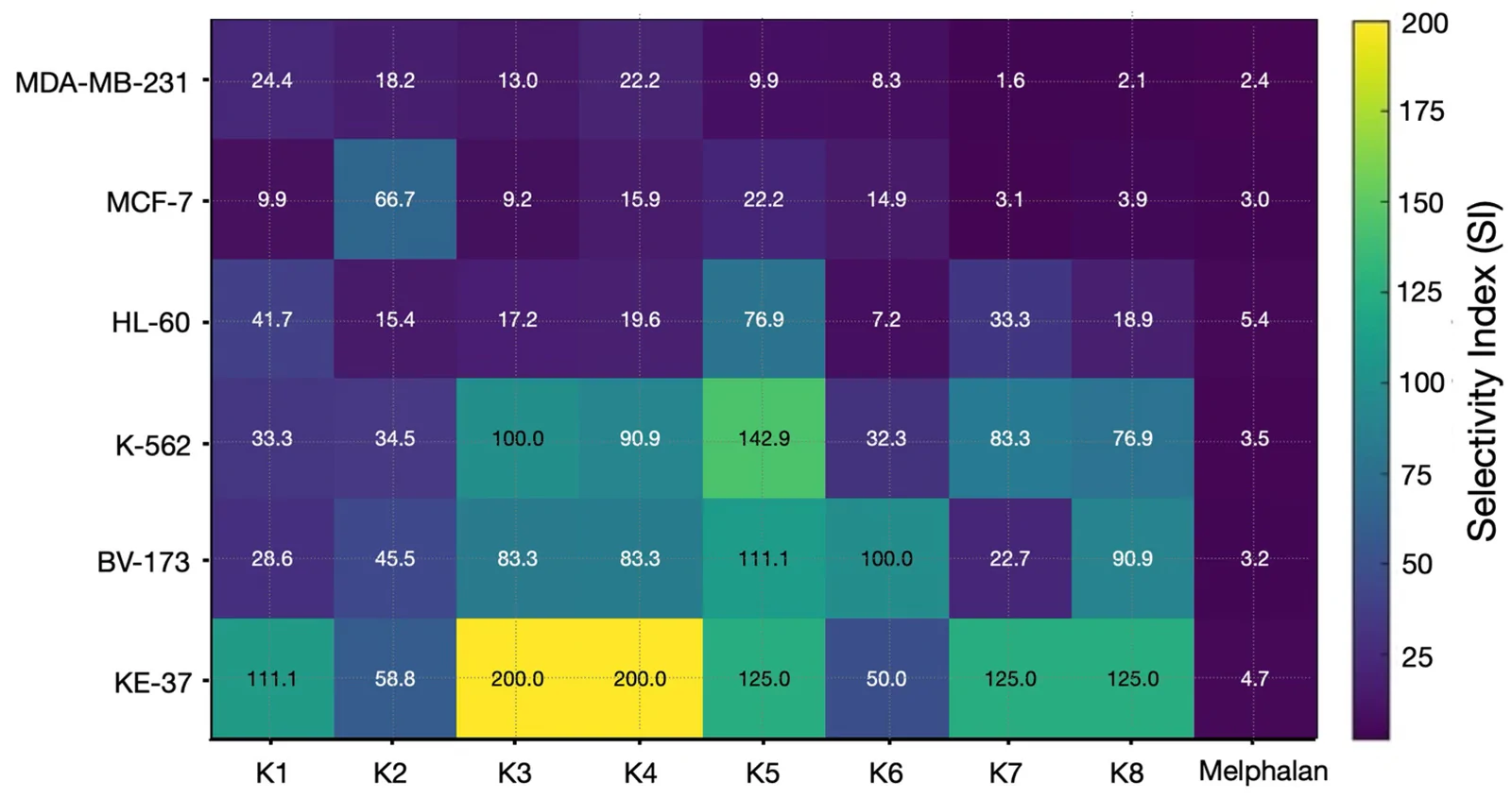
Heatmap of selectivity indices (SIs), highlighting the broader therapeutic window of the newly synthesized hydrazone derivatives relative to melphalan, with highest selectivity observed in leukemic cells.

The ligand efficiencies (LEs; LE = pIC_50_ divided by the number of heavy atoms) and selectivity indices (SIs, SI = IC_50_ in normal CCL-1 cells/IC_50_ in tumor cell lines) were calculated and are presented in Table 4 and Figure 3.

The average LEs of the compounds on leukemic cells range from 0.276 to 0.318, exceeding those observed for breast cancer cells, which range from 0.217 to 0.277. On leukemic cells, the average LEs decrease in the following order: **K5** > **K1** > **K4** > **K3** > **K8** > **K7** > **K2** > **K6**. Importantly, all compounds show no toxicity toward normal CCL-1 cells. The average SIs on leukemic cells range from >38 to >114, compared to average SIs on breast cancer cells ranging from >2 to >42. For leukemic cells, the compounds’ average SIs decrease in the following order: **K5** > **K3** > **K4** > **K8** > **K7** > **K1** > **K6** > **K2**. The highest SI values are observed in SKW-3 cell lines, ranging from >50 to >200, with an average SI of >124.4. For K-562 cell lines, SIs range from >32.3 to >142.9, with an average of 74.3, while BV-173 cells show SIs from >22.7 to >111.1, averaging >70.7. The lowest average SI (>28.8) is observed in HL-60 cells, with values ranging from >15.4 to >76.9.

### 2.4. Structure–Activity Relationships of Chloro-Substituted Salicylaldehyde Benzoylhydrazones

The present series was designed as a focused positional SAR study to examine how chlorine substitution on the salicylaldehyde and benzhydrazide aromatic rings influences the cytotoxic activity and selectivity of salicylaldehyde benzoylhydrazones. Overall, derivatives bearing a 4-chloro substituent on the salicylaldehyde ring (**K1**–**K4**) exhibited superior cytotoxicity compared to the 5-chloro analogs (**K5**–**K8**). Among the benzhydrazide chlorination patterns, substitutions at the 2-, 3-, or 4-position produced modest differences, although ortho- and para-substituted analogs (**K2**, **K4**, **K6**, and **K8**) tended to perform better than meta-substituted compounds (**K3**, **K7**). Notably, **K3** and **K4**, with 3- and 4-chloro substituents in the benzhydrazide of 4-chlorosalicylaldehyde series, consistently ranked among the most potent agents in leukemic lines, with sub-micromolar activity in SKW-3, K-562, and BV-173. Conversely, the meta-substituted 5-chloro-series member **K7** was generally weaker, particularly against the breast carcinoma cell lines. These observations suggest that both the position of chlorination on the salicylaldehyde and benzhydrazide rings contributes to the observed activity differences, potentially through changes in molecular conformation, lipophilicity, hydrogen-bonding capacity, and other physicochemical properties of the hydrazone scaffold.

These findings can be further interpreted in the context of our previous structure–activity studies of salicylaldehyde benzoylhydrazones bearing methoxy, bromo, nitro, and dimethoxy substituents [31,32,33,34,36,37]. Because these compounds were synthesized and evaluated in our laboratory under the same experimental conditions, their cytotoxic profiles provide a directly relevant experimental basis for comparison with the present chloro-substituted series. The present derivatives exhibited comparable or, for selected compounds and leukemia cell lines, greater potency than several of the previously reported analogs, indicating that chlorine substitution represents an additional effective means of modulating the biological activity of this scaffold.

As demonstrated in Figure 3, the selectivity indices (SIs) calculated relative to the non-malignant murine fibroblast line CCL-1 were consistently high across the tested cancer cell lines. SI values exceeded 10 for most compounds and cell lines and, in several cases, exceeded 100, particularly in the SKW-3 leukemia model. Because the IC_50_ values for CCL-1 exceeded the highest concentration tested, these SI values represent lower-bound estimates rather than precise measures of absolute selectivity. Nevertheless, the data indicate a favorable differential in vitro cytotoxicity toward the malignant cell lines relative to CCL-1. For example, in SKW-3 cells, **K3** and **K4** showed SI values above 200, whereas melphalan yielded an SI of approximately 5. Similarly, in MCF-7 cells, **K2** and **K5** exhibited SI values above 60 and 70, respectively, compared with an SI of approximately 3 for melphalan. Overall, the chloro-substituted hydrazones displayed more favorable in vitro selectivity profiles than the reference compound melphalan across the tested malignant cell lines.

Collectively, our findings demonstrate that the newly synthesized chlorosalicylaldehyde benzoylhydrazones possess potent and selective cytotoxic activity, with a pronounced preference for leukemic over epithelial cancer cell lines. The antitumor spectrum of the compounds in the series appears to vary depending on the location of halogen substitution, with 4-chlorosalicylaldehyde derivatives generally outperforming their 5-chloro counterparts. The high-selectivity indices indicate reduced toxicity toward normal murine fibroblasts as compared to melphalan, suggesting a more favorable therapeutic profile. Collectively, these findings indicate that the substitution pattern, rather than chlorine incorporation alone, contributes substantially to the cytotoxic profile of this scaffold. The present series therefore provides a focused positional SAR framework that can be further expanded in future studies through the introduction of additional substituent classes and systematic evaluation of their effects on cytotoxicity and selectivity.

### 2.5. In Silico Evaluation of the Intermolecular Interactions Within the ATP Binding Site at the Human cAbl TK and Chloro-Substituted Salicylaldehyde Benzoylhydrazones

The newly synthesized compounds **K1**–**K8** exhibited pronounced cytotoxic activity against the BV-173 and K-562 leukemia cell lines (Table 3), both of which are BCR::ABL1-positive and represent established models of chronic myeloid leukemia (CML). The BCR::ABL1 fusion encodes a constitutively active ABL1 tyrosine kinase that acts as a principal oncogenic driver in these cells, promoting leukemic proliferation and survival through persistent kinase signaling [56,57,58]. Consequently, ABL1 represents a clinically validated therapeutic target. Based on the pronounced sensitivity of the BCR::ABL1-positive leukemia cells, we investigated whether ABL1 tyrosine kinase (ABL1 TK) could represent a potential molecular target of the chloro-substituted salicylaldehyde benzoylhydrazones. To explore their potential binding mode, molecular docking studies were performed using the human c-Abl tyrosine kinase domain.

**Figure 4:**
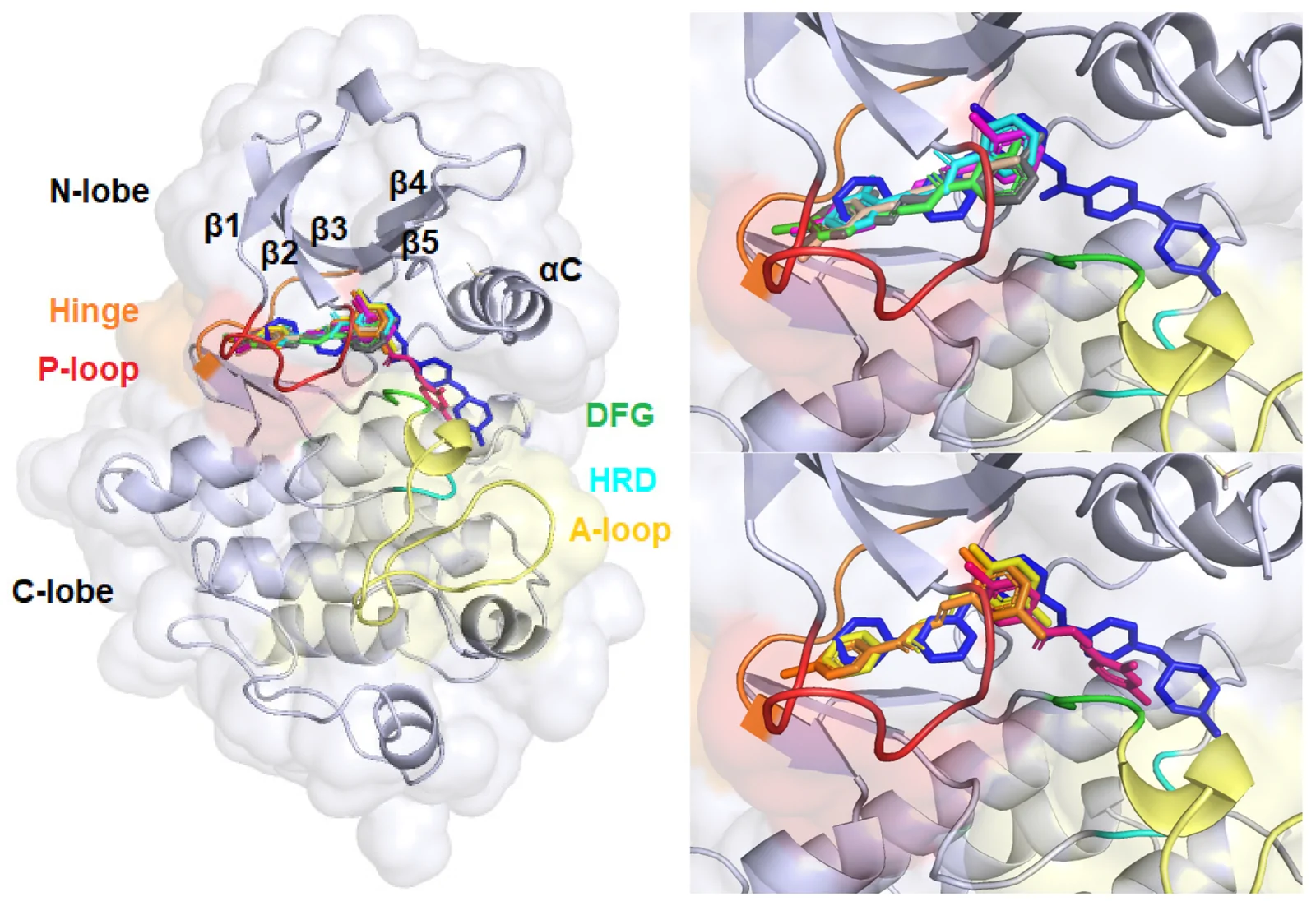
Binding site of human Abelson tyrosine kinase (PDB ID: 2HYY, [58]) with hinge, P-loop, A-loop, DFG, and HRD regions color-coded in orange, red, yellow, green, and cyan, respectively. The N- and C-lobes, αC helix, and β1–β5 elements are indicated. Superimposed docking poses of **K1**–**K8** are shown in cyan, magenta, green, red, wheat (**K5** with salicylaldehyde ring, ring A) oriented toward the entrance formed by P-loop and hinge, **K5A**), yellow (for **K5** flipped pose with phenyl ring, ring B, oriented toward the entrance, **K5B**), purple, dark gray, and orange, respectively; the reference drug Imatinib is shown in dark blue.

The ABL1 kinase domain is organized into two lobes that delimit the ATP-binding cleft. The smaller N-terminal lobe comprises the β1–β5 strands and the αC-helix, whereas the larger C-terminal lobe is predominantly α-helical; the two lobes are connected by the hinge region, which accommodates the conformational movements associated with opening and closing of the kinase domain [56]. The ATP-binding region comprises several conserved structural elements, including the glycine-rich P-loop, hinge region, αC-helix, HRD motif (His361–Arg362–Asp363), and DFG motif (Asp381–Phe382–Gly383) within the activation loop (A-loop) (Figure 4) [56,57]. These structural elements define the architecture of the ATP-binding pocket and provide the principal framework for ligand accommodation.

Several conserved residues contribute to the structural organization of the kinase domain. Within the αC-helix, Glu286 forms a salt bridge with Lys271 on the β3-strand, while Met290 represents RS3 of the regulatory spine, a conserved hydrophobic arrangement associated with kinase conformational organization. The catalytic HRD is located within the catalytic loop, whereas the DFG sequence marks the N-terminal region of the activation loop and contributes to its conformational organization. The aspartate residue of the DFG motif is also involved in coordination of the magnesium ion required for ATP-dependent catalysis [56,57].

The crystallographic complex of ABL1 with imatinib provides a structural reference for the organization of these elements (Figure 4). In this complex, the DFG motif adopts the DFG-out conformation, while the αC-helix remains in the C-in orientation. This arrangement is accompanied by positioning of Met290 within the regulatory spine and maintenance of the Glu286–Lys271 salt bridge. The P-loop, located at the entrance to the ATP-binding region, is particularly relevant to the present docking analysis because its position defines part of the ligand-accessible environment.

Molecular docking calculations were performed for the newly synthesized chloro-substituted salicylaldehyde benzoylhydrazones (**K1**–**K8**) against the ATP-binding site (ATP-BS) of ABL1 tyrosine kinase (ABL1 TK), as described in Section 4. The ChemPLP scores of the top-ranked poses fell within a relatively narrow range (64.21–72.14) (64.21–72.14) (Table 5), indicating broadly comparable predicted binding affinities across the series, in agreement with the similar levels of cytotoxic activity observed against the BCR::ABL1-positive cell lines (Table 3).

Superposition of the top-ranked docking poses is shown in Figure 4. Seven of the eight chloro-substituted hydrazones predominantly occupied the region extending from the P-loop toward the hinge region. In **K1**–**K3**, **K5A**, and **K6**–**K7**, the salicylaldehyde moiety (ring A) was oriented toward the pocket entrance formed by the P-loop and hinge, whereas the benzoyl ring (ring B) extended toward the gatekeeper residue Thr315 at the opposite end of the hinge region. **K8** adopted an inverted orientation relative to this predominant binding mode. **K5** was represented by two nearly isoenergetic poses, **K5A** (ChemPLP = 66.02), which followed the predominant orientation, and **K5B** (ChemPLP = 67.00), which adopted an orientation similar to **K8**. In contrast, **K4** exhibited a distinct binding mode, occupying the region surrounding the αC-helix, HRD motif, and activation loop (A-loop), with its benzoyl ring oriented toward Thr315 (Figure 4).

**Figure 5:**
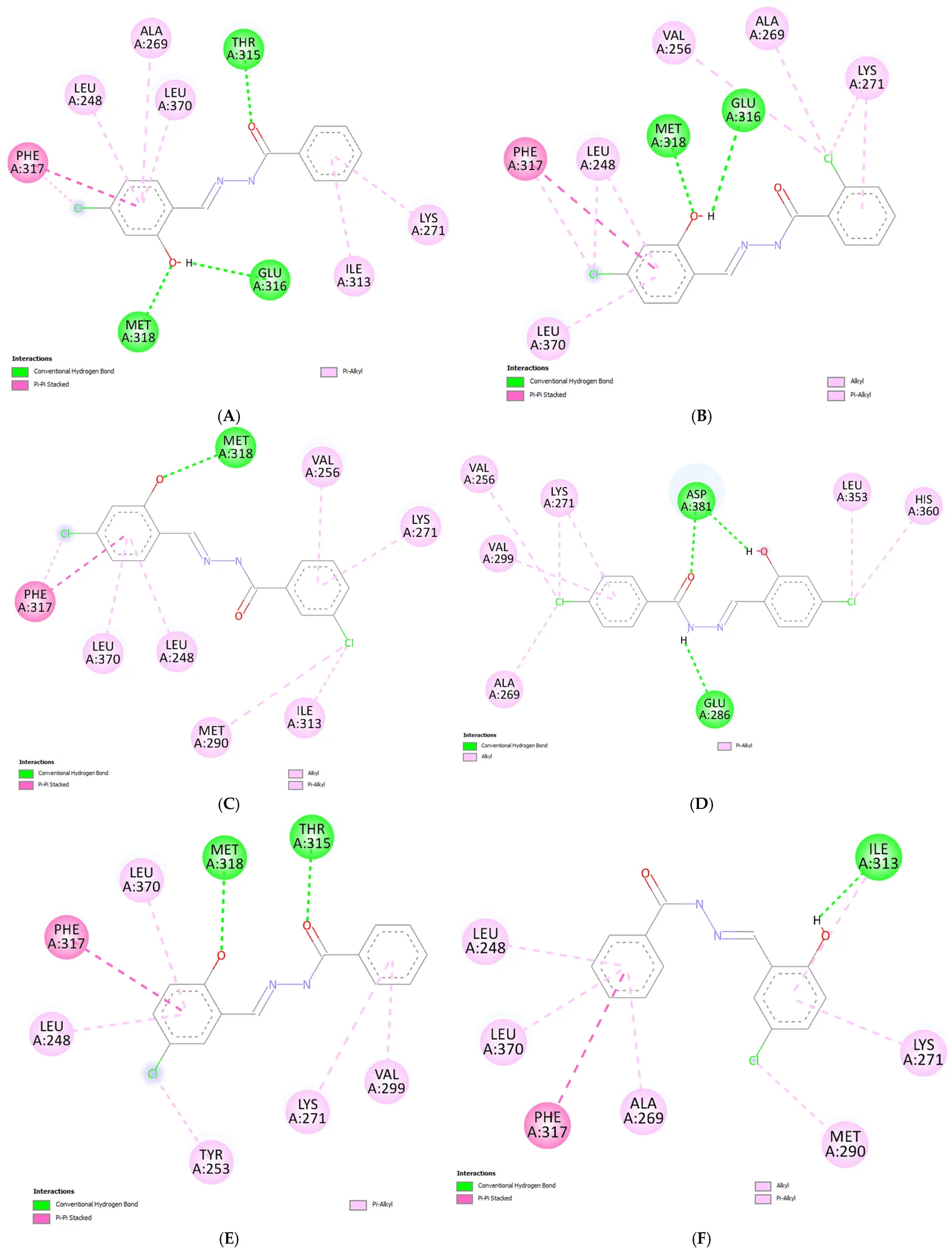

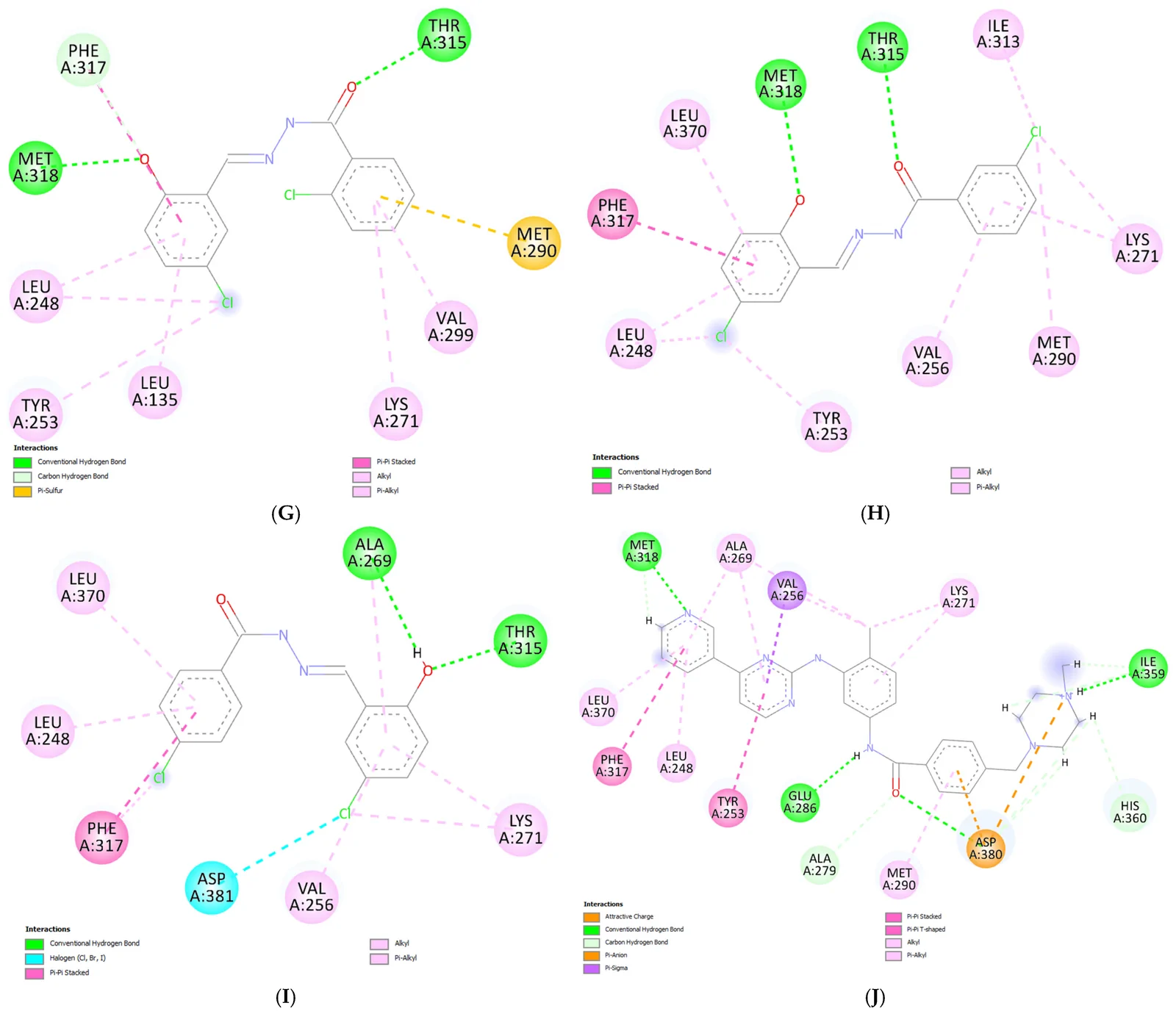
Intermolecular interactions of the top-ranked poses of the studied chloro-substituted salicylaldehyde hydrazones within the ATP-binding site of Abl TK: (**A**) **K1**; (**B**) **K2**; (**C**) **K3**; (**D**) **K4**; (**E**) **K5A**, with the salicylaldehyde ring oriented toward the P-loop; (**F**) **K5B**, with the benzoyl ring oriented toward the P-loop; (**G**) **K6**; (**H**) **K7**; (**I**) **K8**; and (**J**) imatinib. Two-dimensional interaction maps were generated using BIOVIA Discovery Studio Visualizer (v21.1.0.20298; Dassault Systèmes, San Diego, CA, USA, 2021) [59].

The compounds adopting the predominant orientation, with ring A directed toward the entrance of the ATP-BS (**K1**–**K3**, **K5A**, and **K6**–**K7**), displayed broadly similar intermolecular interaction patterns within the ABL1 binding site. These interactions included a hydrogen bond between the hydroxyl oxygen of ring A and the backbone amide N–H of the hinge residue Met318, together with π–π stacking between the salicylaldehyde ring and Phe317 of the hinge region (Figure 5). Additional hydrogen bonding between the hydroxyl hydrogen of ring A and the backbone carbonyl oxygen of Glu316 was observed for **K1** and **K2**. The hydrazone linker, particularly its carbonyl oxygen in **K1**, **K5A**, **K6**, and **K7**, also participated in hydrogen bonding with the hydroxyl group of the gatekeeper residue Thr315. The chlorine substituent on ring A established aromatic or hydrophobic contacts with Phe317 in **K1**–**K3**, whereas in **K5A** and **K6**–**K7** it interacted with P-loop residues Leu248 and Tyr253. Chlorine substituents on ring B formed additional contacts with residues lining the ATP-binding pocket, including Val256 in the P-loop, Ala269 and Lys271 in the β3-strand, Met290 in the αC-helix, and Ile313 in β5, depending on the substitution pattern. In **K6**, Met290 additionally formed a π–sulfur interaction with ring B. The complexes adopting the predominant binding orientation were further stabilized by multiple van der Waals contacts involving Leu248, Val256, Ala269, Leu270, Lys271, Val299, and Ile313.

Compounds **K5B** and **K8** adopted flipped binding orientations, with ring B directed toward the pocket entrance. This rearrangement resulted in the loss of the characteristic hydrogen-bonding interactions with Met318 and Glu316 observed in the predominant binding mode, although π–π stacking with Phe317 was retained. In **K5B**, the hydroxyl hydrogen of ring A formed a hydrogen bond with the backbone carbonyl oxygen of Ile313 (β5). In **K8**, the hydroxyl oxygen and hydrogen atoms of ring A formed hydrogen bonds with Thr315 and Ala269, respectively. Notably, the chlorine atom of **K8** formed a halogen interaction with the backbone carbonyl oxygen of Asp381 within the DFG motif and additional contacts with Lys271. The flipped poses were further stabilized by van der Waals interactions involving Leu248, Ala269, Leu370, Val256, Lys271, and Ile313 (Figure 5).

The top-ranked pose of **K4** adopted a distinct binding mode within the region encompassing the αC-helix, HRD motif, and activation loop (A-loop), with the benzoyl ring oriented toward Thr315 (Figure 4). Three hydrogen-bonding interactions involving residues within the ATP-binding region were observed. The side-chain carboxylate of Glu286 (αC-helix) interacted with the hydrazone NH group, while the side-chain carboxylate of Asp381 (DFG motif) interacted with the hydroxyl group of ring A. In addition, the backbone amide NH of Asp381 formed a hydrogen bond with the linker carbonyl oxygen (Figure 5). The chlorine substituents on rings A and B formed additional contacts with Leu353, His360 (HRD motif), Val256, Ala269, and Lys271. Further stabilization was provided by van der Waals contacts involving Lys271 and Val299.

For comparison, the binding mode of the reference type II ATP-competitive inhibitor imatinib is characterized by a well-established network of interactions within the ABL1 kinase ATP-binding site (PDB ID: 2HYY) [58]. A key interaction involves a hydrogen bond between the backbone amide NH of the hinge residue Met318 and the pyridine nitrogen of imatinib. The hydroxyl group of the gatekeeper residue Thr315 additionally interacts with the anilino NH group of the inhibitor. Two further hydrogen bonds involve residues associated with kinase regulation as follows: the side-chain carboxylate of Glu286 in the αC-helix interacts with the linker amide NH group, while the backbone amide NH of Asp381 in the DFG motif forms a hydrogen bond with the linker carbonyl oxygen. An ionic interaction is also established between the positively charged piperazine nitrogen and the negatively charged side chain of Asp381. In addition, Asp381 contributes to ligand stabilization through a π–anion interaction with the benzamide aromatic ring. Aromatic interactions further stabilize the complex, including π–π stacking between Phe317 in the hinge region and the pyridine ring, and a T-shaped π–π interaction between Tyr253 of the P-loop and the pyrimidine moiety of imatinib.

## 3. Discussion

The incorporation of halogen atoms into biologically active molecules has become one of the most successful strategies in contemporary medicinal chemistry. Halogenation not only modifies the electronic properties of aromatic systems but also influences molecular lipophilicity, conformational preferences, metabolic stability, membrane permeability, and interactions with biological macromolecules [38,41]. Among the halogens, chlorine occupies a particularly important position because of its optimal combination of atomic size, electronegativity, polarizability, and synthetic accessibility. Consequently, chlorine-containing compounds account for a substantial proportion of approved pharmaceuticals and lead compounds currently under development [38,42]. In many cases, replacement of a hydrogen atom by chlorine markedly improves biological activity without requiring extensive modification of the parent molecular scaffold [38,41]. The position of the chlorine atom has an important influence. Even compounds possessing identical molecular formulae may therefore exhibit significantly different physicochemical properties, pharmacokinetic behavior, and biological activities.

The computational analyses confirmed that all synthesized chloro-derivatives display lead-like characteristics, including low molecular weight, moderate lipophilicity, and full compliance with Lipinski’s rules. Comparison between the 4-chloro- and 5-chloro-salicylaldehyde cores reveals minimal differences in logP values, suggesting that chloro substitution on the salicylaldehyde ring exerts a limited influence on overall lipophilicity relative to modifications on the hydrazide phenyl ring. These variations may be explained by differences in intramolecular interactions, steric effects, and molecular conformation. For instance, ortho substitution can promote intramolecular hydrogen bonding, potentially reducing effective polarity, while meta and para substitutions allow for a more extended conformation and slightly greater hydrophobic surface exposure.

The cytotoxic activity of eight newly synthesized chlorosalicylaldehyde benzoylhydrazone derivatives was assessed in epithelial breast cancer models, human leukemic cell lines, and the murine non-malignant fibroblast line CCL-1. A consistent trend was observed in which leukemic cells were substantially more sensitive to the hydrazone derivatives than the epithelial cancer lines. Across the leukemia panel, IC_50_ values generally fell within the low-micromolar or sub-micromolar range, with several derivatives showing substantially greater potency than melphalan. In the BCR::ABL1-positive K-562 and BV-173 cell lines, selected compounds also exhibited markedly lower IC_50_ values than imatinib. SKW-3 cells, derived from T-cell prolymphocytic leukemia, were particularly responsive. Most compounds exhibited sub-micromolar IC_50_ values (0.5–0.9 µM), with only **K2** (1.7 µM) and **K6** (2.0 µM) showing slightly higher values. Compounds **K3** and **K4** were the most active, achieving IC_50_ values of 0.5 µM—representing more than a 40-fold increase in potency relative to melphalan (21.4 µM). Comparable activity was observed in chronic myeloid leukemia (CML)-derived K-562 and BV-173 cells.

Because the compounds considered in the present study and the previously reported methoxy-, bromo-, nitro-, and dimethoxy-substituted derivatives were synthesized and evaluated in our laboratory under the same experimental conditions, their cytotoxic profiles can be directly compared [31,33,34,36,37]. The comparative data reveal a distinct activity pattern associated with chlorination within the salicylaldehyde benzoylhydrazone scaffold. The previously reported derivatives exhibited considerable variation in cytotoxic potency depending on both the nature and position of the substituents [31,33,34,36,37]. For example, the 3-methoxysalicylaldehyde derivative showed moderate activity against HL-60 and K-562 cells, with IC_50_ values of 11.3 and 27.0 µM, respectively, whereas introduction of a 4-hydroxy group on the benzoyl ring in the corresponding increased activity against several leukemia models, including BV-173 (IC_50_ = 7.8 µM) [31,36]. The corresponding 5-bromosalicylaldeyde-4-hydroxybenzoyl derivative exhibited greater potency, with IC_50_ values of 3.14, 2.83, and 2.02 µM against HL-60, K-562 and BV-173 cells, respectively [36]. In contrast, the 5-nitrosalicylaldehyde derivative showed particularly strong activity against breast cancer cell lines, with IC_50_ values of 0.25 ± 0.03 µM against MCF-7 and 2.8 µM against MDA-MB-231 cells [34,36]. Similarly, the 5-methoxysalicylaldehyde derivative exhibited pronounced activity against MCF-7 cells, with an IC_50_ value of 0.91 µM [31,34,36]. More recently, the dimethoxy-substituted series demonstrated strong activity against leukemia cells, with selected derivatives showing IC_50_ values of 1.5–2.8 µM against K-562, 1.4–2.7 µM against BV-173, and 0.5–2.7 µM against SKW-3 cells [37].

Against this background, several compounds from the present chloro-substituted series exhibited comparable or, in selected cases, greater potency than the previously reported analogs. In particular, **K5** showed IC_50_ values of 0.7 ± 0.1 and 0.9 ± 0.1 µM against K-562 and BV-173 cells, respectively, while **K3** and **K4** reached 0.5 ± 0.1 µM against SKW-3 cells. These values place the most active chloro-substituted derivatives among the most potent compounds identified within this series of salicylaldehyde benzoylhydrazone anseries. Importantly, however, the comparison demonstrates that chlorination does not uniformly enhance cytotoxicity across all cancer models. For example, the 5-chloro derivatives **K7** and **K8** showed considerably weaker activity against MDA-MB-231 and MCF-7 cells than several previously reported nitro- and dimethoxy-substituted analogs, while retaining low-micromolar or sub-micromolar activity against several leukemia cell lines. Thus, the effect of chlorination appears to depend strongly on its position within the molecular scaffold and on the cellular context.

Collectively, these comparisons extend the existing structure–activity relationships of salicylaldehyde benzoylhydrazones and indicate that chlorine substitution, particularly within the 4-chlorosalicylaldehyde series, can favor cytotoxic activity against selected leukemia models. This observation is consistent with the broader literature on acylhydrazones, in which relatively small structural modifications can substantially alter cytotoxic potency and selectivity [60,61,62]. For example, Congiu and Onnis reported 2-hydroxybenzylidene acylhydrazones with submicromolar cytotoxic activity against several tumor cell lines, further illustrating how modification of aromatic substituents can produce pronounced differences in biological response [62]. Taken together, the present findings expand the structure–activity landscape of salicylaldehyde benzoylhydrazones by showing that positional chlorine substitution can be used to modulate cytotoxic potency, particularly toward leukemia cells. Nevertheless, these results represent an in vitro assessment of cytotoxic potential, and further studies addressing molecular targets, cellular mechanisms of action, selectivity in broader panels of non-malignant cells, and in vivo efficacy are required to determine the translational potential of these compounds.

The selectivity indices (SIs) calculated relative to the non-malignant murine fibroblast line CCL-1 were generally high across the series. For most cancer cell lines, the SI values exceeded 10, and several values were greater than 100, particularly for the SKW-3 leukemic model. Because the IC_50_ values for CCL-1 exceeded the highest concentration tested (>100 µM), these SI values represent lower-bound estimates rather than exact quantitative measures of selectivity. Nevertheless, the data indicate favorable in vitro selectivity of the chloro-substituted hydrazones toward the malignant cell lines relative to CCL-1. Direct quantitative assessment of absolute normal-cell cytotoxicity remains limited, and further evaluation using appropriate human non-malignant cell models will be required to establish the selectivity profile more comprehensively.

The high in vitro SI values observed for the chloro-substituted hydrazones contrast with the lower selectivity observed for melphalan. Across all malignant cell lines, the chloro-hydrazone derivatives outperformed melphalan both in terms of selectivity and potency. Melphalan displayed IC_50_ values in the tens of micromolar range in leukemic cells and >30 µM in epithelial cells, whereas multiple hydrazone derivatives achieved single-digit or sub-micromolar IC_50_ values. The superior cytotoxic activity of the hydrazone analogs was most outstanding in the leukemic lines, where they were 20- to 40-fold more potent than melphalan. Even in the more resistant MDA-MB-231 breast cancer line, several hydrazones (**K1**, **K2**, and **K4**) demonstrated stronger activity than melphalan. In terms of selectivity, melphalan lacked a substantial therapeutic margin, with SI estimates generally below 5, in contrast to the hydrazone derivatives, whose values consistently exceeded 10 and frequently surpassed 50.

Molecular docking studies of the novel chloro-substituted salicylaldehyde benzoylhydrazones within the ATP-binding site (ATP-BS) of Abl TK revealed a predominant preference for the region formed by the hinge and P-loop. Most compounds (**K1**–**K3**, **K5A**, and **K6**–**K7**) adopted a common orientation in which the salicylaldehyde moiety (ring A) was directed toward the pocket entrance, whereas the benzoyl ring (ring B) extended toward the gatekeeper residue Thr315. The following two key interactions involving the hinge region contributed to stabilization of these complexes: a hydrogen bond between the hydroxyl group of ring A and Met318, and a π–π stacking interaction with Phe317. In addition, hydrogen bonding between the linker carbonyl oxygen and Thr315 was observed for derivatives **K1**, **K5A**, **K6**, and **K7**. Collectively, these interactions are consistent with a predicted type II ATP-binding mode involving the hinge region and the gatekeeper residue Thr315 [63,64].

Compound **K8**, although occupying a similar region of the ATP-BS, adopted a flipped orientation with the benzoyl ring directed toward the pocket entrance. This binding mode was primarily stabilized through hydrogen bonds involving the salicylaldehyde hydroxyl group and residues Thr315 and Ala269, together with a halogen interaction between the chlorine substituent of ring A and Asp381 of the DFG motif. The absence of the canonical Met318 interaction suggests a distinct, potentially non-classical binding mode within the ATP-binding site.

In contrast, derivative **K4** occupied the deeper back-pocket region formed by the αC-helix, HRD motif, and activation loop (A-loop). Stabilization of this complex was driven by hydrogen bonding with two catalytically important residues, Glu286 from the αC-helix and Asp381 from the DFG motif. These interactions may indicate an alternative binding arrangement within the ATP-binding site.

The chlorine substituents on both the salicylaldehyde (ring A) and benzoyl (ring B) moieties contributed additional stabilizing contacts that influenced ligand orientation and localization within the ATP-BS. The most pronounced effect was observed for the ring A chlorine atom of **K8**, which participated in a halogen interaction with Asp381, whereas the ortho-chlorine substituent of ring B in compound **K6** showed minimal involvement in stabilizing interactions. Overall, the substitution pattern and positional arrangement of chlorine atoms appear to influence ligand positioning within the ATP-BS, directing the compounds either toward the entrance region formed by the hinge and P-loop or toward the deeper pocket defined by the αC-helix, HRD motif, and A-loop. The predicted poses are consistent with a type II ATP-binding mode involving Met318 and Thr315 for **K1**–**K3**, **K5A**, and **K6**–**K7**, whereas **K4** and **K8** adopt alternative predicted binding arrangements involving Asp381 and Glu286 or Asp381 and Thr315, respectively [64,65]. These docking results provide a structural basis for considering ABL1 as a potential molecular target; however, they should be regarded as suggestive evidence of possible ligand engagement rather than as evidence of ABL1 inhibition or proof that ABL1 mediates the observed cytotoxicity.

Several limitations of the present study should be acknowledged. First, the structural series represents a focused positional SAR, and the 3-position of the salicylaldehyde ring was not investigated. Extension of the series to include this substitution pattern, together with additional electron-donating and electron-withdrawing substituents, would provide a broader basis for defining the structure–activity relationships of this scaffold. Second, although the compounds exhibited favorable predicted drug-like properties, their limited aqueous solubility and less favorable predicted oral bioavailability indicate that further structural optimization will be required to improve their pharmacokinetic characteristics. Third, the biological evaluation was based on an in vitro cytotoxicity assay, and therefore the observed activity does not by itself establish the molecular mechanism responsible for the leukemia-selective effects. Similarly, the ABL1 docking results identify plausible binding modes but provide only suggestive evidence of potential ABL1 engagement and do not establish ABL1 inhibition as the mechanism of cytotoxicity. Broader studies using additional non-malignant human cell models, biochemical target-validation assays, mechanistic cellular studies, and ultimately in vivo pharmacokinetic and efficacy investigations will therefore be necessary to determine the therapeutic potential of these compounds. Nevertheless, the present findings identify chloro-substituted salicylaldehyde benzoylhydrazones as a structurally tunable series with pronounced and selective cytotoxicity toward leukemia cell lines and provide a basis for further optimization and mechanistic investigation.

## 4. Materials and Methods

### 4.1. Materials

All reagents and solvents used in the study were of analytical grade. 4-Chlorosalicylaldehyde, 5-chlorosalicylaldehyde, 2-chlorobenzhydrazide, 3-chlorobenzhydrazide, 4-chlorobenzhydrazide, and 96% ethanol were purchased from Merck/Sigma-Aldrich (Darmstadt, Germany). Elemental compositions for carbon, hydrogen, and nitrogen were determined using a Euro EA 3000-Single elemental analyzer (EuroVector SpA, Milan, Italy). FT-IR spectra were collected with a Nicolet iS10 spectrometer equipped with a Smart iTR adapter. NMR characterization was performed on a Bruker Avance 400 spectrometer (Rheinstetten, Germany) using DMSO-d_6_ as the deuterated solvent and TMS as the internal standard. Chemical shifts (δ) are expressed in ppm, and coupling constants (J) are reported in Hz. The observed multiplicities are abbreviated as s (singlet), d (doublet), t (triplet), and m (multiplet).

### 4.2. Synthesis and Characterization

4-Chloro- or 5-chlorosalicylaldehyde (0.01 mol) was dissolved in 96% ethanol (75 mL), while benzhydrazide or the corresponding 2-, 3-, or 4-chlorobenzhydrazide (0.01 mol) was separately dissolved in 50% aqueous ethanol (75 mL). The two solutions were combined gradually under continuous stirring. The resulting mixtures were maintained at 50–60 °C with stirring for 90–120 min and subsequently cooled to room temperature. The reaction mixtures were then left undisturbed for 168 h to allow crystallization of the products. The resulting crystals were isolated by filtration and dried under vacuum for 48 h.

#### 4.2.1. 4-Chlorosalicylaldehyde benzoylhydrazone (K1)

Yield: 92% (2.52 g); color: pale yellow; m.p.: 220–222 °C; IR (ν cm^−1^): 3204 (OH), 3152 (NH), 1646 (C=O), 1605 (C=N), 1557 (C-NH). ^1^H NMR (400 MHz, DMSO-d_6_) δ ppm: 7.00 (m, 2H), 7.55 (dd, *J* = 8.2, 6.6 Hz, 2H), 7.62 (m, 2H), 7.94 (m, 2H), 8.64 (s, 1H, CH=N), 11.59 (s, 1H, NH), 12.15 (s, 1H, OH). ^13^C NMR (101 MHz, DMSO-d_6_) δ ppm: 116.65, 118.67, 120.06, 128.12, 129.05, 130.80, 132.52, 133.19, 135.76, 147.09 (CH=N), 158.55, 163.42 (C=O). HR ESI–MS *m*/*z*: 275.0574 [M + H]^+^. Calculated for C_14_H_11_ClN_2_O_2_: C, 61.21; H, 4.04; N, 10.20. Found: C, 61.38; H, 4.14; N, 10.28.

#### 4.2.2. 4-Chlorosalicylaldehyde-2-chlorobenzoylhydrazone (K2)

Yield: 89% (2.75 g); color: pale yellow; m.p.: 211–213 °C; IR (ν cm^−1^): 3202 (OH), 3148 (NH), 1653 (C=O), 1602 (C=N), 1559 (C-NH). ^1^H NMR (400 MHz, DMSO-d_6_) δ ppm: 6.86 (m, 2H), 6.99 (m, 2H), 7.25 (d, *J* = 8.1 Hz, 1H), 7.55 (m, 2H), 8.49 (s, 1H, CH=N), 11.32 (s, 1H, NH), 12.16 (s, 1H, OH). ^13^C NMR (101 MHz, DMSO-d_6_) δ ppm: 116.62, 118.98, 127.65, 128.96, 129.59, 129.95, 131.26, 135.20, 135.95, 142.68, 146.82 (CH=N), 157.65, 162.79 (C=O), 168.76. HR ESI–MS *m*/*z*: 309.0184 [M + H]^+^. Calculated for C_14_H_10_Cl_2_N_2_O_2_: C, 54.39; H, 3.26; N, 9.06. Found: C, 54.67; H, 3.18; N, 9.15.

#### 4.2.3. 4-Chlorosalicylaldehyde-3-chlorobenzoylhydrazone (K3)

Yield: 87% (2.69 g); color: pale yellow; m.p.: 213–215 °C; IR (ν cm^−1^): 3400 (OH), 3168 (NH), 1650 (C=O), 1603 (C=N), 1566 (C-NH). ^1^H NMR (400 MHz, DMSO-d_6_) δ ppm: 7.00 (m, 2H), 7.59 (t, *J* = 8 Hz, 1H), 7.68 (m, 2H), 7.90 (d, *J* = 8 Hz, 1H), 7.99 (t, *J* = 1.9 Hz, 1H), 8.64 (s, 1H, CH=N), 11.46 (s, 1H, NH), 12.20 (s, 1H, OH). ^13^C NMR (101 MHz, DMSO-d_6_) δ ppm: 116.64, 118.71, 120.10, 126.98, 127.81, 130.57, 131.08, 132.29, 133.81, 135.28, 135.89, 147.25 (CH=N), 158.53, 161.93 (C=O). HR ESI–MS *m*/*z*: 309.0183 [M + H]^+^. Calculated for C_14_H_10_Cl_2_N_2_O_2_: C, 54.39; H, 3.26; N, 9.06. Found: C, 54.62; H, 3.30; N, 9.22.

#### 4.2.4. 4-Chlorosalicylaldehyde-4-chlorobenzoylhydrazone (K4)

Yield: 91% (2.81 g); color: pale yellow; m.p.: 256–258 °C; IR (ν cm^−1^): 3380 (OH), 3184 (NH), 1645 (C=O), 1605 (C=N), 1594 (C-NH). ^1^H NMR (400 MHz, DMSO-d_6_) δ ppm: 7.00 (m, 2H), 7.64 (m, 3H), 7.97 (m, 2H), 8.64 (s, 1H, CH=N), 11.51 (s, 1H, NH), 12.19 (s, 1H, OH). ^13^C NMR (101 MHz, DMSO-d_6_) δ ppm: 116.65, 118.69, 120.08, 129.15, 130.06, 130.67, 131.98 135.83, 137.32, 147.16 (CH=N), 158.54, 162.30 (C=O). HR ESI–MS *m*/*z*: 309.0183 [M + H]^+^. Calculated for C_14_H_10_Cl_2_N_2_O_2_: C, 54.39; H, 3.26; N, 9.06. Found: C, 54.59; H, 3.41; N, 9.19.

#### 4.2.5. 5-Chlorosalicylaldehyde benzoylhydrazone (K5)

Yield: 93% (2.55 g); color: pale yellow; m.p.: 223–224 °C; IR (ν cm^−1^): 3418 (OH), 3216 (NH), 1646 (C=O), 1603 (C=N), 1575 (C-NH). ^1^H NMR (400 MHz, DMSO-d_6_) δ ppm: 6.97 (d, *J* = 8.8 Hz, 1H), 7.33 (dd, *J* = 8.8, 2.7 Hz, 1H), 7.55 (m, 2H), 7.62 (m, 1H), 7.68 (d, *J* = 2.7 Hz, 1H), 7.95 (m, 2H), 8.63 (s, 1H, CH=N), 11.30 (s, 1H, NH), 12.20 (s, 1H, OH). ^13^C NMR (101 MHz, DMSO-d_6_) δ ppm: 118.71, 121.18, 123.44, 128.08, 128.16, 129.03, 131.25, 132.52, 133.21, 146.30 (CH=N), 156.51, 163.45 (C=O). HR ESI–MS *m*/*z*: 275.0573 [M + H]^+^. Calculated for C_14_H_11_ClN_2_O_2_: C, 61.21; H, 4.04; N, 10.20. Found: C, 61.42; H, 3.90; N, 10.20.

#### 4.2.6. 5-Chlorosalicylaldehyde-2-chlorobenzoylhydrazone (K6)

Yield: 90% (2.78 g); color: pale yellow; m.p.: 188–189 °C; IR (ν cm^−1^): 3220 (OH), 3191 (NH), 1646 (C=O), 1606 (C=N), 1593 (C-NH). ^1^H NMR (400 MHz, DMSO-d_6_) δ ppm: 6.97 (s, 1H), 7.22 (m, 1H), 7.33 (d, *J* = 8.8, 1H), 7.53 (m, 3H), 7.69 (m, 1H), 8.48 (s, 1H, CH=N), 11.04 (s, 1H, NH), 12.21 (s, 1H, OH). ^13^C NMR (101 MHz, DMSO-d_6_) δ ppm: 118.70, 121.18, 126.63, 127.63, 129.01, 129.54, 130.92, 131.27, 135.19, 141.88, 146.11 (CH=N), 155.70, 162.88 (C=O), 168.87. HR ESI–MS *m*/*z*: 309.0184 [M + H]^+^. Calculated for C_14_H_10_Cl_2_N_2_O_2_: C, 54.39; H, 3.26; N, 9.06. Found: C, 54.68; H, 3.39; N, 9.28.

#### 4.2.7. 5-Chlorosalicylaldehyde-3-chlorobenzoylhydrazone (K7)

Yield: 87% (2.68 g); color: pale yellow; m.p.: 213–215 °C; IR (ν cm^−1^): 3380 (OH), 3210 (NH), 1636 (C=O), 1604 (C=N), 1563 (C-NH). ^1^H NMR (400 MHz, DMSO-d_6_) δ ppm: 6.97 (d, *J* = 8.8 Hz, 1H), 7.33 (dd, *J* = 8.7, 2.7 Hz, 1H), 7.59 (t, *J* = 7.9 Hz, 1H), 7.69 (m, 2H), 7.91 (m, 1H), 8.00 (t, 1H), 8.64 (s, 1H, CH=N), 11.19 (s, 1H, NH), 12.25 (s, 1H, OH). ^13^C NMR (101 MHz, DMSO-d_6_) δ ppm: 118.72, 121.19, 123.50, 127.01, 127.84, 131.07, 131.41, 132.31, 133.81, 135.25, 146.53 (CH=N), 156.50, 162.03 (C=O). HR ESI–MS *m*/*z*: 309.0184 [M + H]^+^. Calculated for C_14_H_10_Cl_2_N_2_O_2_: C, 54.39; H, 3.26; N, 9.06. Found: C, 54.70; H, 3.38; N, 8.91.

#### 4.2.8. 5-Chlorosalicylaldehyde-4-chlorobenzoylhydrazone (K8)

Yield: 90% (2.78 g); color: pale yellow; m.p.: 258–260 °C; IR (ν cm^−1^): 3350 (OH), 3210 (NH), 1635 (C=O), 1593 (C=N), 1563 (C-NH). ^1^H NMR (400 MHz, DMSO-d_6_) δ ppm: 6.96 (d, *J* = 8.7 Hz, 1H), 7.33 (dd, *J* = 8.8, 2.7 Hz, 1H), 7.64 (m, 2H), 7.69 (d, *J* = 2.7 Hz, 1H), 7.97 (m, 2H), 8.63 (s, 1H, CH=N), 11.23 (s, 1H, NH), 12.24 (s, 1H, OH). ^13^C NMR (101 MHz, DMSO-d_6_) δ ppm: 118.72, 121.18, 123.48, 127.94, 129.14, 130.09, 131.35, 131.96, 137.35, 146.43 (CH=N), 156.50, 162.39 (C=O). HR ESI–MS *m*/*z*: 309.0184 [M + H]^+^. Calculated for C_14_H_10_Cl_2_N_2_O_2_: C, 54.39; H, 3.26; N, 9.06. Found: C, 54.57; H, 3.12; N, 8.93.

### 4.3. Cell Lines and Culture Conditions

The antitumor profile of the synthesized compounds was evaluated using a panel of human cancer cells comprising HL-60 (acute promyelocytic leukemia), K-562 (chronic myeloid leukemia), BV-173 (BCR::ABL1-positive leukemia), SKW-3 (T-cell prolymphocytic leukemia), MCF-7 (estrogen receptor-positive breast adenocarcinoma), and MDA-MB-231 (triple-negative breast adenocarcinoma), together with the non-malignant CCL-1 mouse fibroblast cell line. All cell lines were obtained from the German Collection of Microorganisms and Cell Cultures (DSMZ GmbH, Braunschweig, Germany) and maintained according to established cell culture procedures and the supplier’s recommendations (Leibniz Institute DSMZ–German Collection of Microorganisms and Cell Cultures. Human and Animal Cell Lines Catalog. Available online at https://www.dsmz.de/; accessed on 12 August 2026). Cells were cultured in RPMI-1640 medium supplemented with 10% heat-inactivated fetal bovine serum (FBS), 2 mM L-glutamine, and penicillin-streptomycin (100 U/mL penicillin and 100 µg/mL streptomycin), at 37 °C in a humidified atmosphere containing 5% CO_2_. Cell cultures were routinely maintained under aseptic conditions and subcultured as required to sustain exponential growth.

### 4.4. Mosmann’s MTT Test for Assessing Cell Viability

Cytotoxic activity of the chloro-substituted salicylaldehyde benzoylhydrazone derivatives was determined using the validated Mosmann MTT assay for cell viability [65]. Exponentially growing cells were seeded in 96-well plates of 3 × 10^5^ cells/mL for suspension leukemia cell lines and 1.5 × 10^5^ cells/mL for adherent epithelial cell lines, using 100 µL of cell suspension per well, corresponding to 3 × 10^4^ and 1.5 × 10^4^ cells per well, respectively. Following a 24 h pre-incubation period, the cells were exposed for 72 h to 10 serially decreasing concentrations of the tested compounds, starting from a final concentration of 100 µM in the wells. Stock solutions of the compounds were prepared in DMSO at 10 mM (10,000 µM) and subsequently diluted to the respective treatment concentrations. Ten serially diluted concentrations, starting from 100 µM, were tested for each compound, covering a concentration range extending well below 1 µM. Appropriate solvent-treated controls were included. Each concentration was tested in at least eight technical replicate wells, and the complete experiment was independently performed three times.

At the end of the 72 h treatment period, filter-sterilized MTT solution (5 mg/mL in PBS) was added to each well, followed by incubation for 4 h to allow the formation of insoluble purple formazan crystals. The resulting formazan was dissolved in isopropanol containing 5% formic acid, and absorbance was measured at 550 nm using a Labexim LMR-1 microplate reader. Absorbance values were corrected against MTT and isopropanol blanks, and cell viability was expressed as a percentage relative to the corresponding untreated control, defined as 100%. Concentration–response curves were fitted by nonlinear regression using a four-parameter logistic (4PL) dose–response inhibition model with variable slope in GraphPad Prism^®^ v8, with the bottom parameter left unconstrained. Three independent experiments were performed, with each treatment concentration tested in six replicate wells within each experiment. IC_50_ values are presented as the mean ± SD of the three independent experiments.

### 4.5. In Silico Evaluation of Pharmacokinetic Parameters

The investigated compounds were modeled using BIOVIA Discovery Studio Visualizer (v21.1.0.20298; Dassault Systèmes, San Diego, CA, USA, 2021). Molecular descriptors applied in the QSPR models [43,44,45] were computed with MDL QSAR (v2.2.0.0.446; MDL Information Systems, Inc., 2004, San Leandro, CA, USA) [66].

### 4.6. Molecular Docking Analysis

The computational analysis of the newly synthesized chloro-substituted salicylaldehyde benzoylhydrazones was conducted using a workflow adapted from our previously described procedure [48]. Initial molecular models of the investigated compounds were generated and prepared using Discovery Studio Visualizer v21.1.0.20298 [59]. Molecular docking was subsequently carried out with the human Abelson tyrosine kinase (ABL TK) using the crystallographic structure deposited under PDB ID 2HYY [58]. Docking calculations were performed with GOLD v.5.2.2 (CCDC Ltd., Cambridge, UK) [67], employing the ChemPLP scoring function. During the docking calculations, the protein structure was kept rigid, whereas ligand flexibility was allowed. The docking region was defined around the co-crystallized imatinib molecule, including all protein residues located within 6 Å of the reference ligand. To assess the performance of the docking setup, imatinib was removed from the crystal structure and subsequently re-docked under the same conditions. The experimentally observed and predicted imatinib binding poses showed an RMSD of 0.3868 Å, supporting the ability of the applied protocol to reproduce the crystallographic binding orientation.

## 5. Conclusions

In this work, we examined the anticancer potential of a series of chloro-substituted salicylaldehyde benzoylhydrazones featuring chlorine atoms on both aromatic rings. The obtained results allow several key conclusions to be drawn.

The synthesized chloro-substituted hydrazones possess favorable molecular features and exhibit properties consistent with lead-like and drug-like behavior. Our findings indicate that the newly synthesized chlorosalicylaldehyde benzoylhydrazones exhibit potent and selective in vitro cytotoxic activity, particularly against the tested leukemia cell lines. The anticancer profile of the series appears to depend on the position of halogen substitution, with 4-chlorosalicylaldehyde derivatives consistently showing superior activity relative to the 5-chloro analogs. High-selectivity indices indicate lower cytotoxicity toward normal murine fibroblasts relative to the tested cancer cell lines and, compared with melphalan, support a more favorable in vitro selectivity profile. The combination of tunable structural features, potent activity against hard-to-treat leukemias, and improved selectivity highlights this hydrazone class as a promising direction for future anticancer drug development.

Given the pronounced activity of the compounds against BCR::ABL1-positive chronic myeloid leukemia cell lines BV-173 and K-562, human cAbl tyrosine kinase was investigated as a potential molecular target. Docking studies suggested that the compounds can be accommodated within the ABL1 ATP-binding site and identified plausible ligand–residue interactions that may contribute to their binding. These findings provide a structural rationale for considering ABL1 as a potential molecular target; however, they do not establish ABL1 inhibition as the mechanism underlying the observed cytotoxicity. Further experimental studies are required to confirm ABL1 target engagement and to elucidate the molecular mechanisms responsible for the observed cytotoxic activity.

## Figures and Tables

**Table 1:** Structures of the designed choro-substituted salicylaldehyde benzoylhydrazones.

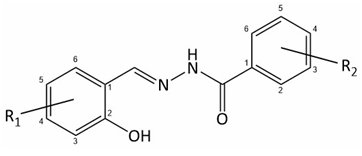	**ID**	**R_1_**	**R_2_**
	**K1**	4-Cl	H
	**K2**	4-Cl	2-Cl
	**K3**	4-Cl	3-Cl
	**K4**	4-Cl	4-Cl
	**K5**	5-Cl	H
	**K6**	5-Cl	2-Cl
	**K7**	5-Cl	3-Cl
	**K8**	5-Cl	4-Cl

**Table 2:** Predicted physicochemical and pharmacokinetic profiles of the chloro-substituted salicylaldehyde benzoylhydrazones.

Property	K1	K2	K3	K4	K5	K6	K7	K8
Mw g/mol	274.70	309.15	309.15	309.15	274.70	309.15	309.15	309.15
pK_a_	7.22	7.31	7.35	7.33	7.73	7.83	7.87	7.85
f_A_	0.60	0.55	0.53	0.54	0.32	0.27	0.25	0.26
logP	2.95	3.46	3.48	3.41	2.93	3.46	3.40	3.40
logD_7.4_	2.55	3.11	3.15	3.07	2.76	3.32	3.27	3.27
PSA Å^2^	61.69	61.69	61.69	61.69	61.69	61.69	61.69	61.69
FRB	4	4	4	4	4	4	4	4
HBDs	2	2	2	2	2	2	2	2
HBAs	3	3	3	3	3	3	3	3
R5	0	0	0	0	0	0	0	0
S	Moderate	Moderate	Moderate	Moderate	Moderate	Moderate	Moderate	Moderate
GI absorp	High	High	High	High	High	High	High	High
Oral BA	INSATU	INSATU	INSATU	INSATU	INSATU	INSATU	INSATU	INSATU
BA score	0.55	0.55	0.55	0.55	0.55	0.55	0.55	0.55
BBB permeab	Yes	Yes	Yes	Yes	Yes	Yes	Yes	Yes
CYP inhib	1A2, 2C9	1A2, 2C19, 2C9	1A2, 2C19, 2C9	1A2, 2C19, 2C9	1A2, 2C9	1A2, 2C19, 2C9	1A2, 2C19, 2C9	1A2, 2C19, 2C9
P-gp substr	No	No	No	No	No	No	No	No
Drug likeness	Yes	Yes	Yes	Yes	Yes	Yes	Yes	Yes
Lead likeness	Yes *	Yes *	Yes *	Yes *	Yes *	Yes *	Yes *	Yes *
SA	2.38	2.47	2.44	2.39	2.32	2.42	2.37	2.34
f_u_	0.057	0.028	0.026	0.033	0.042	0.017	0.019	0.023
CL L/h/kg	1.59	1.06	1.2	1.53	1.23	0.66	0.92	1.12
VDss L/kg	0.206	0.216	0.204	0.206	0.208	0.218	0.206	0.208

Mw—molecular weight, pKa value, f_A_—fraction of the ionized molecules at pH 7.4, logP—partition coefficient, logD_7.4_—distribution coefficient at pH 7.4, PSA—polar surface area, FRB—count of free rotatable bonds, HBDs—hydrogen bond donors, HBAs—hydrogen bond acceptors, R5—count of the violations from Lipinski’s Rule of 5, S—water solubility, GI absorp—gastrointestinal absorption, oral BA—oral bioavailability, INSATU—unsaturation, BA score—bioavailability score, BBB permeab—blood–brain barrier permeability, CYP inhib—inhibition of CYP enzymes, P-gp substr—substrate of P-gp, drug likeness, lead likeness, SA—synthetic accessibility, fu—fraction of the unbound to plasma protein molecules, CL—total clearance, VDss—steady-state volume of distribution; * XLOGP > 3.5.

**Table 3:** Cytotoxicity of the tested hydrazone derivatives (IC_50_, [µM ± SD]) against a panel of human malignant cell lines of different origin and normal murine fibroblast cells (CCL-1).

Compound	MDA- MB-231	MCF-7	HL-60	K-562	BV-173	SKW-3	CCL-1
**K1**	4.1 ± 0.5	10.1 ± 1.2	2.4 ± 0.3	3.0 ± 0.2	3.5 ± 0.4	0.9 ± 0.1	>100
**K2**	5.5 ± 0.4	1.5 ± 0.6	6.5 ± 0.6	2.9 ± 0.2	2.2 ± 0.2	1.7 ± 0.2	>100
**K3**	7.7 ± 0.7	10.9 ± 0.8	5.8 ± 0.2	1.0 ± 0.1	1.2 ± 0.1	0.5 ± 0.1	>100
**K** **4**	4.5 ± 0.3	6.3 ± 0.5	5.1 ± 0.4	1.1 ± 0.2	1.2 ± 0.1	0.5 ± 0.1	>100
**K5**	10.1 ± 1.1	4.5 ± 0.3	1.3 ± 0.2	0.7 ± 0.1	0.9 ± 0.1	0.8 ± 0.1	>100
**K6**	12.1 ± 0.9	6.7 ± 0.4	13.9 ± 1.1	3.1 ± 0.3	1.0 ± 0.1	2.0 ± 0.2	>100
**K7**	62.5 ± 4.6	32.1 ± 3.1	3.0 ± 0.2	1.2 ± 0.1	4.4 ± 0.3	0.8 ± 0.1	>100
**K8**	48.4 ± 2.9	25.4 ± 2.9	5.3 ± 0.5	1.3 ± 0.1	1.1 ± 0.1	0.8 ± 0.1	>100
Melphalan	42.2 ± 3.8	33.7 ± 3.4	18.5 ± 1.8	28.2 ± 2.7	31.3 ± 4.4	21.40 ± 2.6	>100
Imatinib				26.9 ± 2.4 *	21.5 ± 3.3 *		

Standard deviation values were obtained from three independent analyses. * Assessment of imatinib cytotoxicity in CCL-1 cells was considered not applicable, as imatinib is a BCR::ABL1-targeted drug and this particular cellular model is not relevant.

**Table 4:** Ligand efficiencies (*LEs*) and selectivity indices (*SIs*) of the chloro-substituted salicylaldehyde benzoylhydrazones. The cytotoxic activities of the compounds were measured on four leukemia cell lines (HL-60, K-562, BV-173, and SKW-3), one breast ER+ adenocarcinoma cell line (MCF-7), and one TNBC cell line (MDA-MB-231).

Cell Line	K1		K2		K3		K4		K5		K6		K7		K8	
	*LE*	*SI*	*LE*	*SI*	*LE*	*SI*	*LE*	*SI*	*LE*	*SI*	*LE*	*SI*	*LE*	*SI*	*LE*	*SI*
HL-60	0.296	>41.7	0.259	>15.4	0.261	>17.2	0.263	>19.6	0.310	>76.9	0.243	>7.2	0.276	>33.3	0.264	>18.9
K-562	0.291	>33.3	0.277	>34.5	0.300	>100	0.298	>90.9	0.324	>142.9	0.275	>32.3	0.296	>83.3	0.294	>76.9
BV-173	0.287	>28.6	0.283	>45.5	0.296	>83.3	0.296	>83.3	0.318	>111.1	0.300	>100	0.268	>22.7	0.298	>90.9
SKW-3	0.318	>111.1	0.288	>58.8	0.315	>200	0.315	>200	0.321	>125	0.285	>50	0.305	>125	0.305	>125
Avg. leukemia	0.300	>53.7	0.277	>38.5	0.293	>100.1	0.293	>98.5	0.318	>114.0	0.276	>47.4	0.286	>66.1	0.290	>77.9
MCF-7	0.263	>9.9	0.291	>66.7	0.248	>9.2	0.260	>15.9	0.281	>22.2	0.259	>14.9	0.225	>3.1	0.230	>3.9
MDA-MB-231	0.284	>24.4	0.263	>18.2	0.256	>13.0	0.267	>22.2	0.263	>9.9	0.246	>8.3	0.210	>1.6	0.216	>2.1
Avg. breast cancer	0.273	>17.1	0.277	>42.4	0.252	>11.1	0.264	>19.0	0.272	>16.1	0.252	>11.6	0.217	>2.4	0.222	>3
Avg. all cancers	0.290	>41.5	0.277	>39.8	0.279	>70.5	0.284	>72.0	0.303	>81.3	0.270	>35.4	0.263	>44.9	0.268	>53.0
CCL-1	>0.211	-	>0.200	-	>0.200	-	>0.200	-	>0.211	-	>0.200	-	>0.200	-	>0.200	-

**Table 5:** ChemPLP docking scores of the top-ranked poses of the studied Cl-SABH derivatives. For compound **K5**, the following two alternative poses with comparable scores were identified: one with the salicylaldehyde ring (ring A) oriented toward the entrance of the binding pocket (**K5A**), and a second flipped orientation with the benzoyl ring (ring B) directed toward the pocket entrance (**K5B**).

Cmpd	K1	K2	K3	K4	K5A	K5B	K6	K7	K8	Imatinib
ChemPLP	67.20	68.46	68.28	64.21	66.02	67.00	70.34	64.91	72.14	122.38

## Data Availability

The original contributions presented in this study are included in the article/Supplementary Materials. Further inquiries can be directed to the corresponding authors.

## Supplementary Materials

The following supporting information can be downloaded at https://www.mdpi.com/article/10.3390/ph19091387/s1, Figure S1: HR ESI–MS spectra of the compound **K1**; Figure S2: IR spectra of the compound **K1**; Figure S3: ^1^H NMR spectra of the compound **K1**; Figure S4: ^13^C NMR spectra of the compound **K1**; Figure S5: HR ESI–MS spectra of the compound **K2**; Figure S6: IR spectra of the compound **K2**; Figure S7: ^1^H NMR spectra of the compound **K2**; Figure S8: ^13^C NMR spectra of the compound **K2**; Figure S9: HR ESI–MS spectra of the compound **K3**; Figure S10: IR spectra of the compound **K3**; Figure S11: ^1^H NMR spectra of the compound **K3**; Figure S12: ^13^C NMR spectra of the compound **K3**; Figure S13: HR ESI–MS spectra of the compound **K4**; Figure S14: IR spectra of the compound **K4**; Figure S15: ^1^H NMR spectra of the compound **K4**; Figure S16: ^13^C NMR spectra of the compound **K4**; Figure S17: HR ESI–MS spectra of the compound **K5**; Figure S18: IR spectra of the compound **K5**; Figure S19: ^1^H NMR spectra of the compound **K5**; Figure S20: ^13^C NMR spectra of the compound **K5**; Figure S21: HR ESI–MS spectra of the compound **K6**; Figure S22: IR spectra of the compound **K6**; Figure S23: ^1^H NMR spectra of the compound **K6**; Figure S24: ^13^C NMR spectra of the compound **K6**; Figure S25: HR ESI–MS spectra of the compound **K7**; Figure S26: IR spectra of the compound **K7**; Figure S27: ^1^H NMR spectra of the compound **K7**; Figure S28: ^13^C NMR spectra of the compound **K7**; Figure S29: HR ESI–MS spectra of the compound **K8**; Figure S30: IR spectra of the compound **K8**; Figure S31: ^1^H NMR spectra of the compound **K8**; Figure S32: ^13^C NMR spectra of the compound **K8**.
