# Supplementary material for: Design, Synthesis, and Integrated In Silico and In Vitro Evaluation of Chloro-Substituted Salicylaldehyde Benzoylhydrazones as Anticancer Agents
Source: Pharmaceuticals (Basel). 2026 Sep 1;19(9):1387. doi: 10.3390/ph19091387 (PMC13610099; doi:10.3390/ph19091387)
Supplement: Supplementary file 1 [file pharmaceuticals-19-01387-s001.zip › pharmaceuticals-4506256-supplementary.pdf]

Supplementary Material

Design, Synthesis, and Integrated In Silico and In Vitro Evaluation of Chloro-Substituted Salicylaldehyde Benzoylhydrazones as Anticancer Agents

Boryana Nikolova-Mladenova [1,3]*, Rositsa Mihaylova [2,3], Stilyana Kostova[2], Boris Vasilev [1], Irini Doytchinova [1,3], and Mariyana Atanasova [1,3]*

[1] Department of Chemistry, Faculty of Pharmacy, Medical University of Sofia, 2 Dunav Str., 1000 Sofia, Bulgaria
[2] Department of Pharmacology, Pharmacotherapy and Toxicology, Faculty of Pharmacy, Medical University of Sofia, 2 Dunav Str., 1000 Sofia, Bulgaria; rmihaylova@pharmfac.mu-sofia.bg
[3] Centre of Excellence in Informatics and Information and Communication Technologies, 1113 Sofia, Bulgaria
* Correspondence: boriananik@pharmfac.mu-sofia.bg; matanasova@pharmfac.mu-sofia.bg

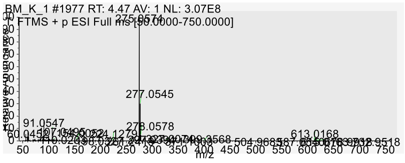

Figure S1. HR ESI–MS spectra of the compound **K1**.

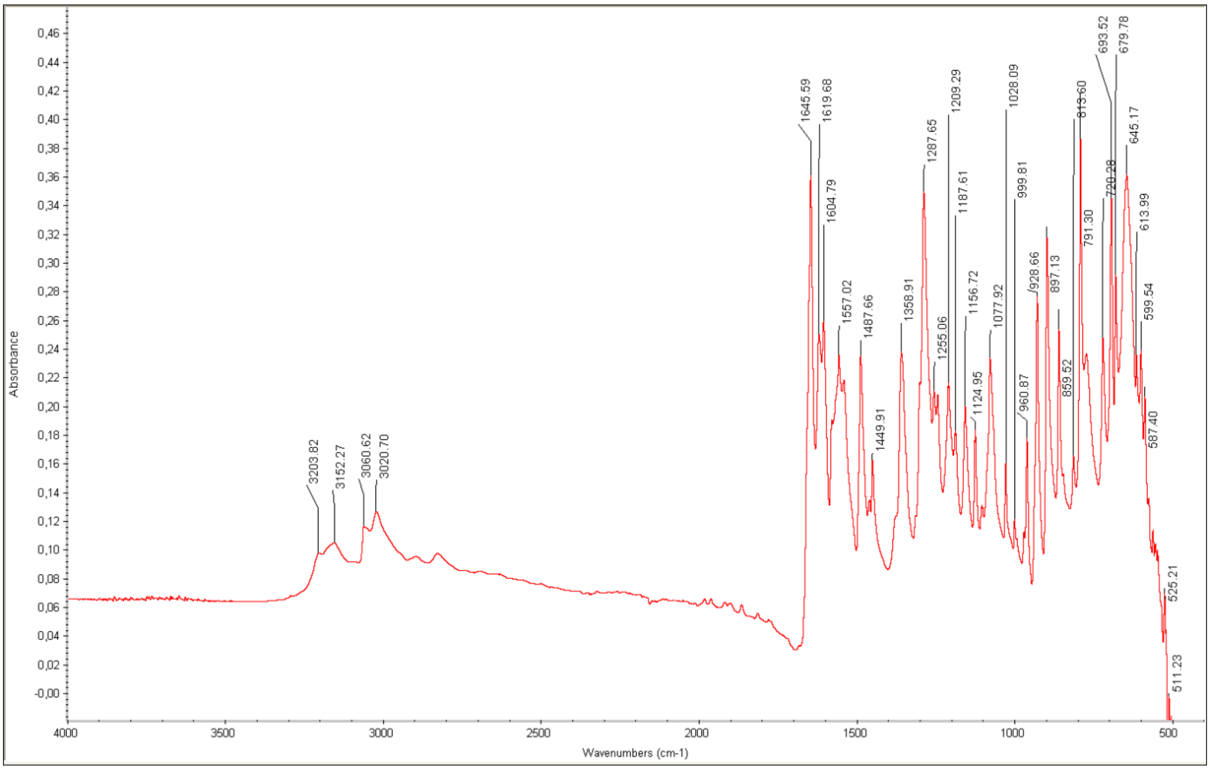

Figure S2. IR spectra of the compound **K1**.

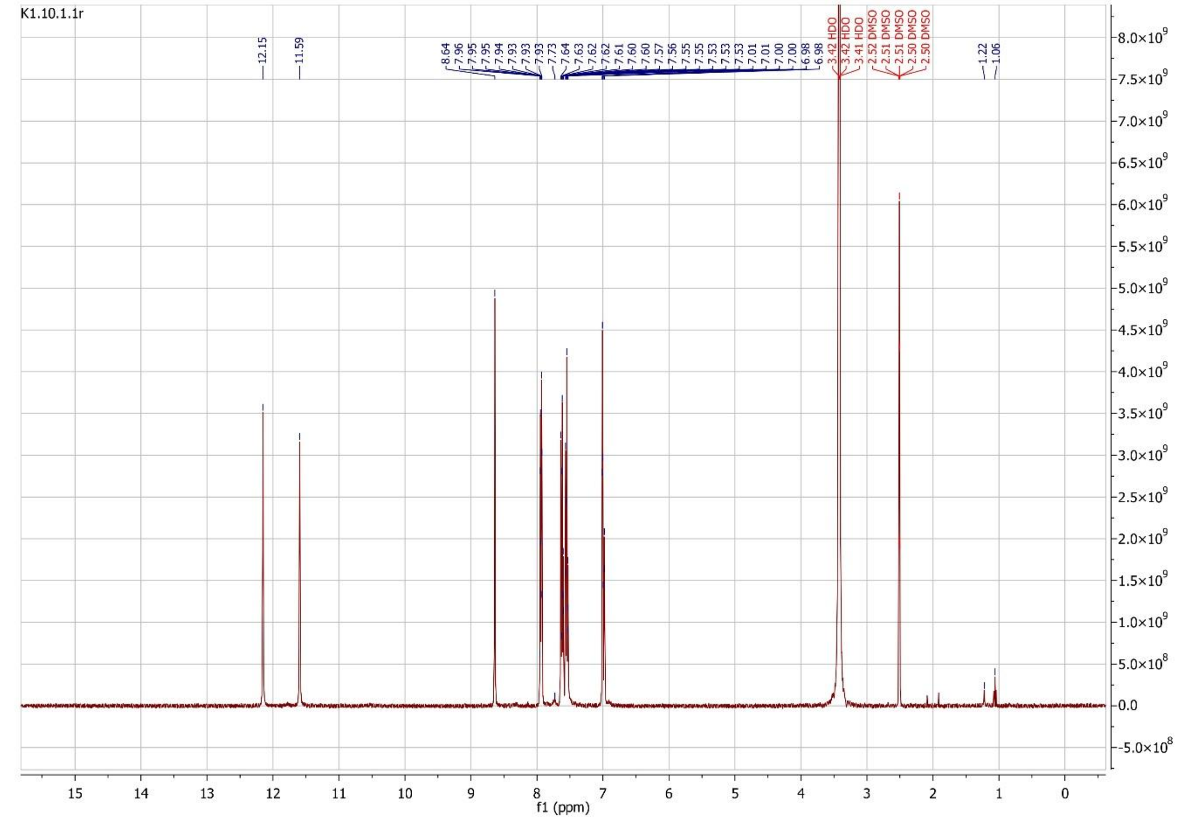

Figure S3. ^{1}H NMR spectra of the compound **K1**.

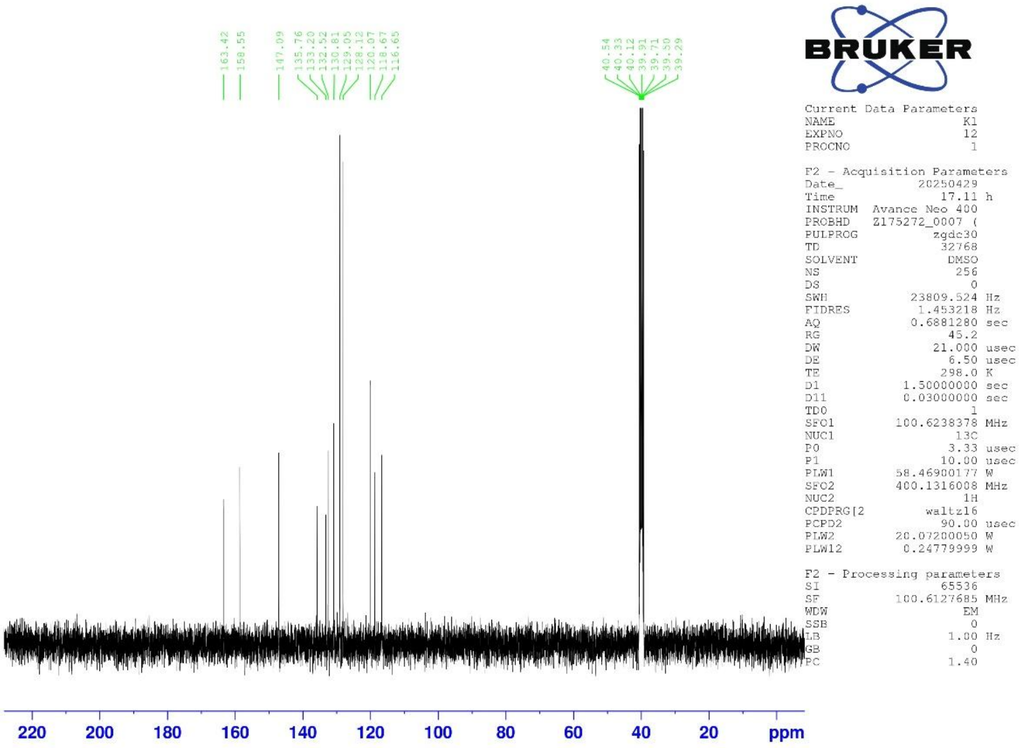

Figure S4. ^{13}C NMR spectra of the compound **K1**.

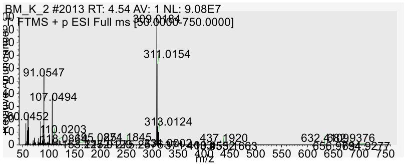

Figure S5. HR ESI–MS spectra of the compound **K2**.

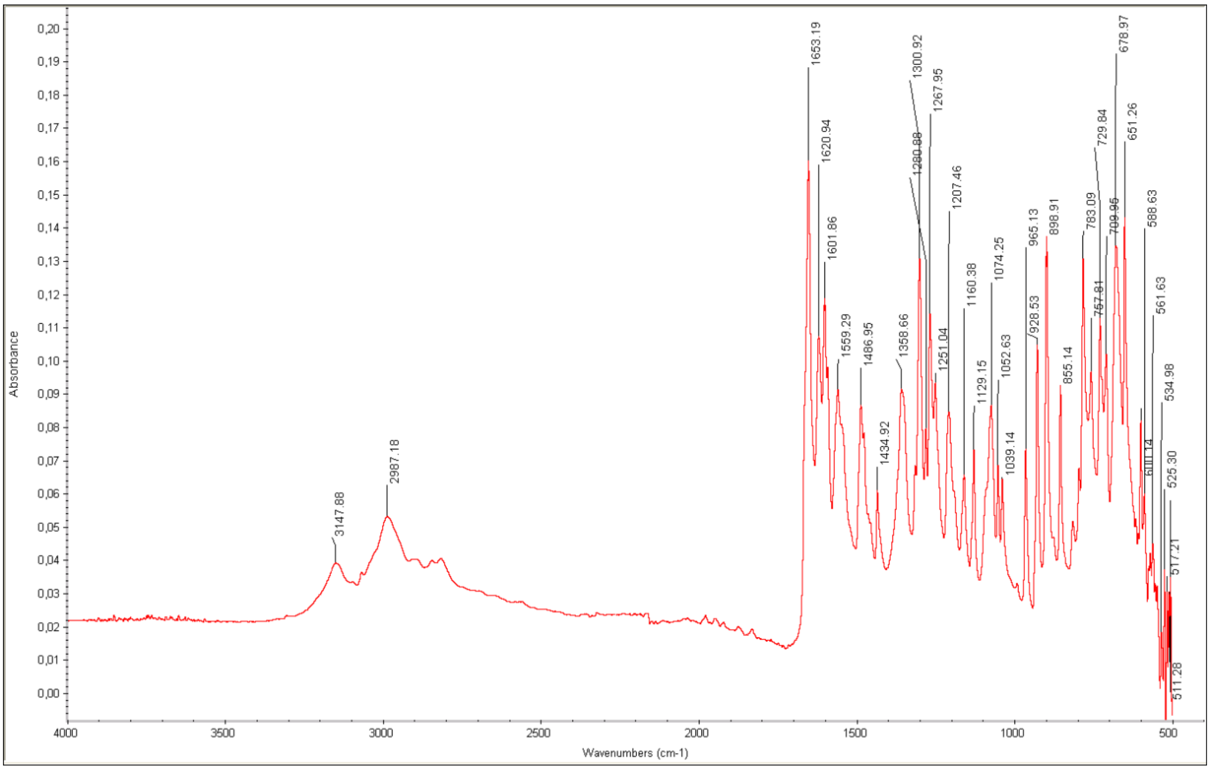

Figure S6. IR spectra of the compound **K2**.

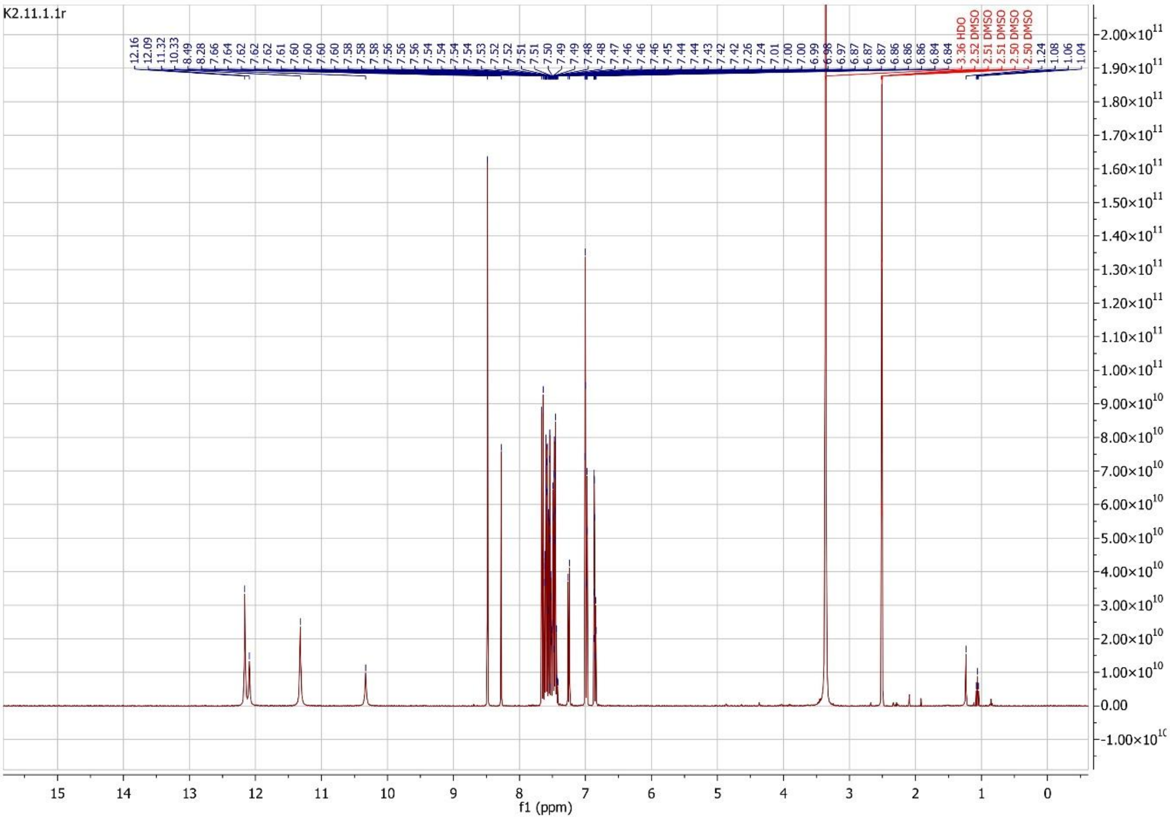

Figure S7. ^{1}H NMR spectra of the compound **K2**.

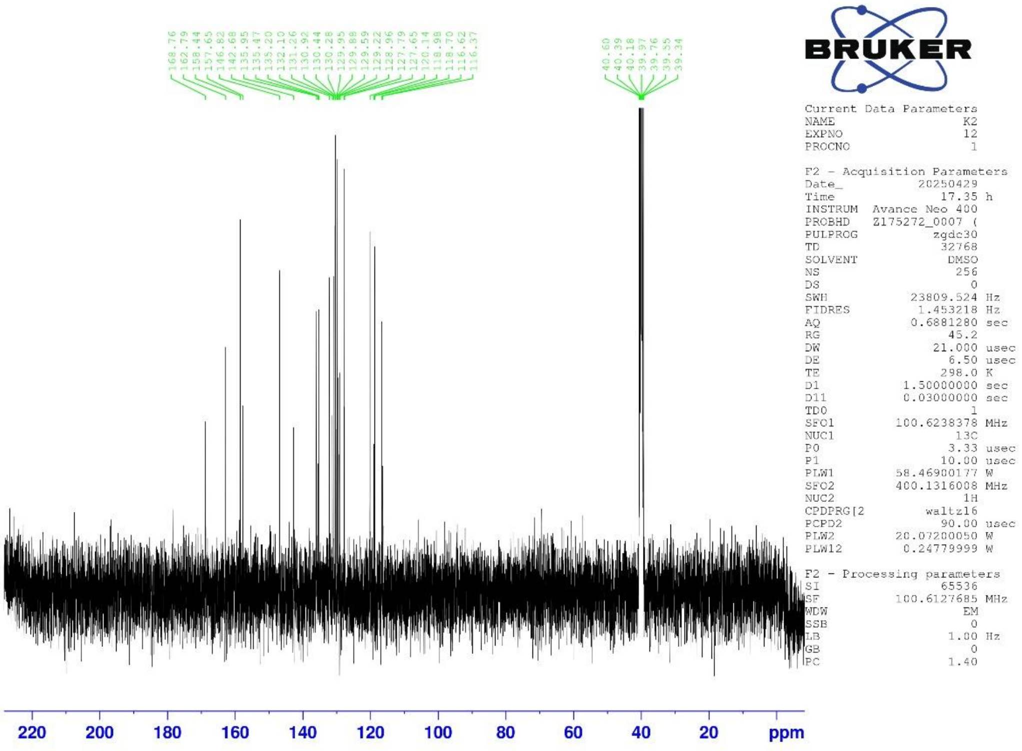

Figure S8. ^{13}C NMR spectra of the compound **K2**.

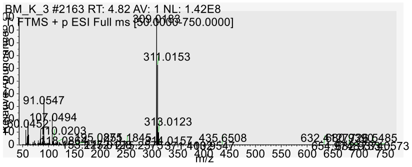

Figure S9. HR ESI–MS spectra of the compound **K3**.

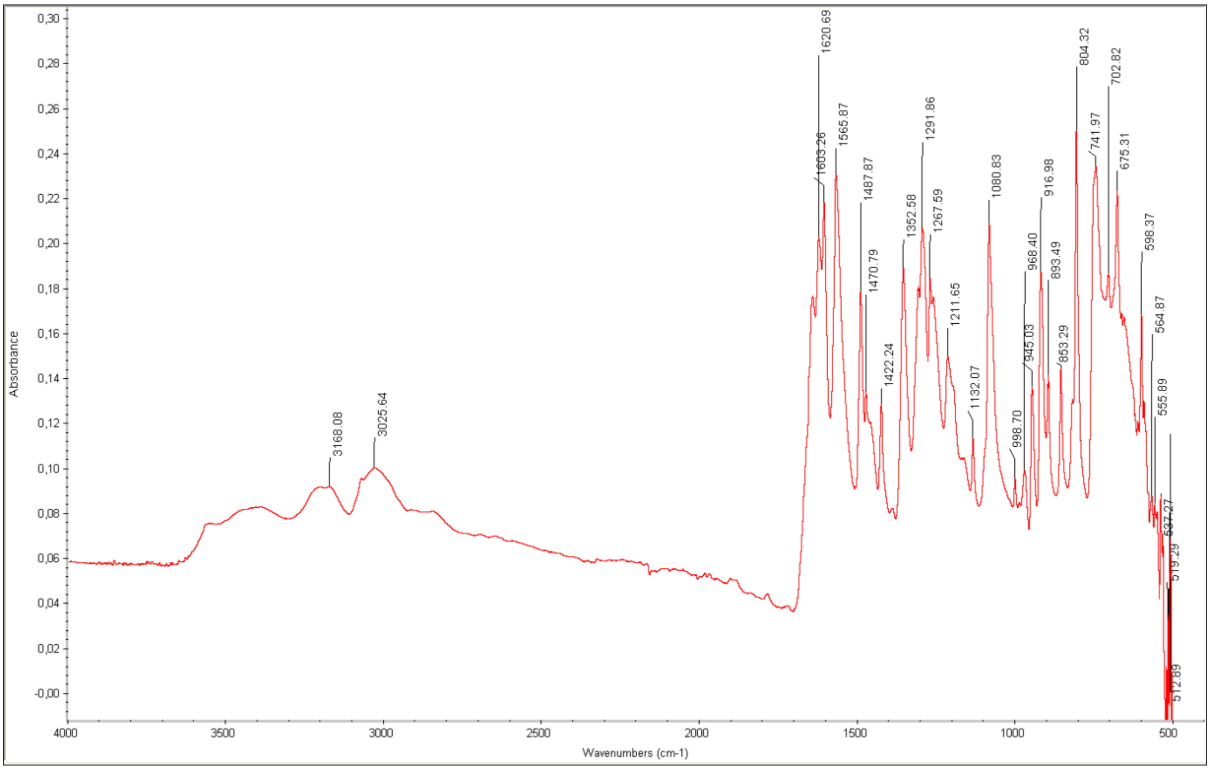

Figure S10. IR spectra of the compound **K3**.

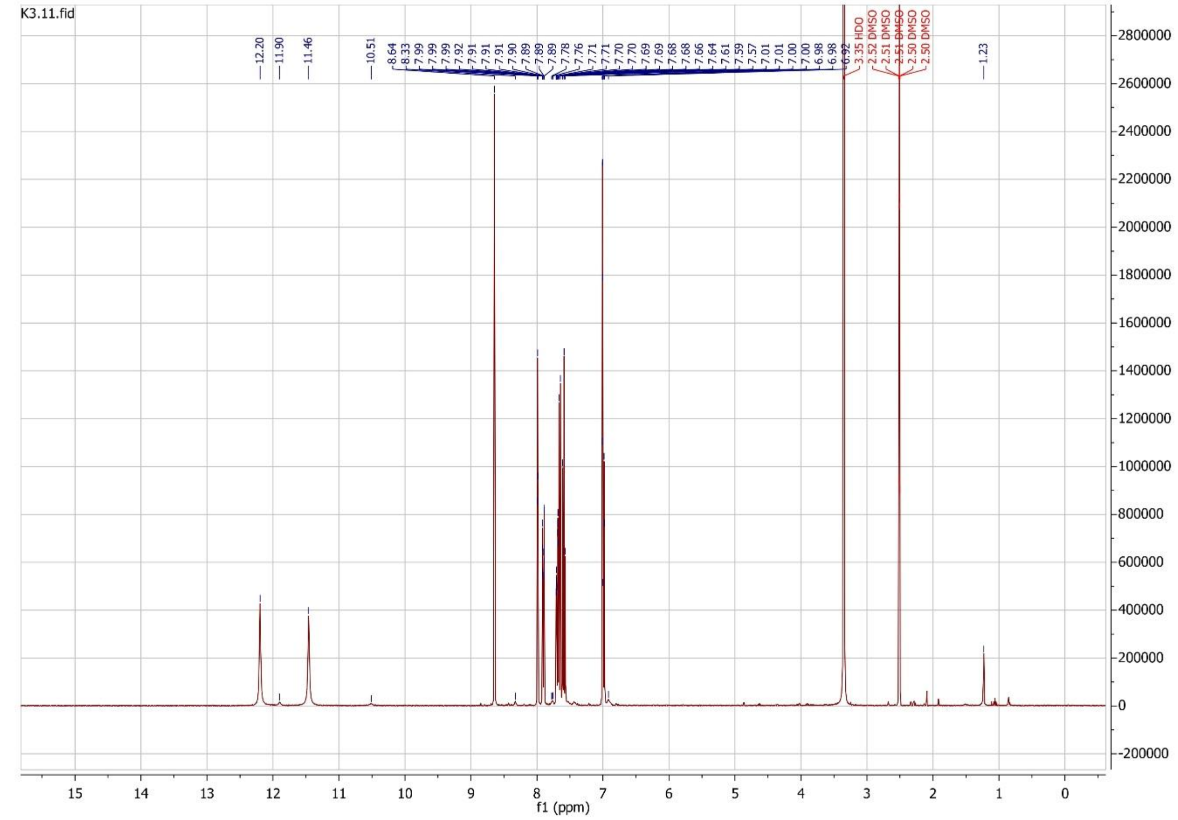

Figure S11. ^{1}H NMR spectra of the compound **K3**.

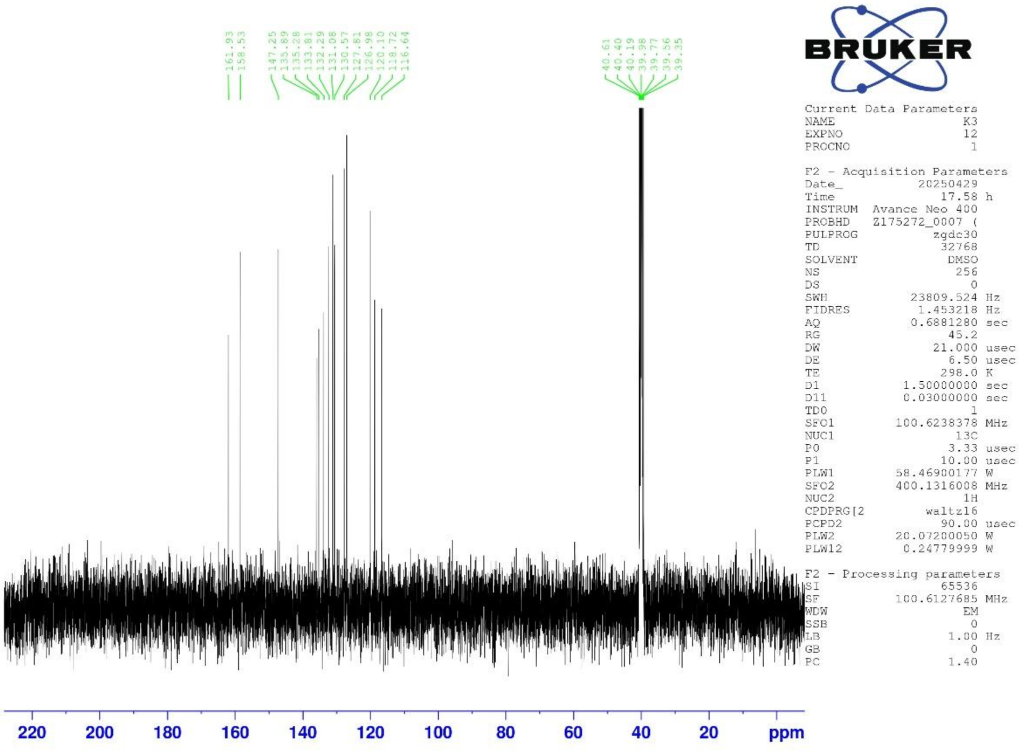

Figure S12. ^{13}C NMR spectra of the compound **K3**.

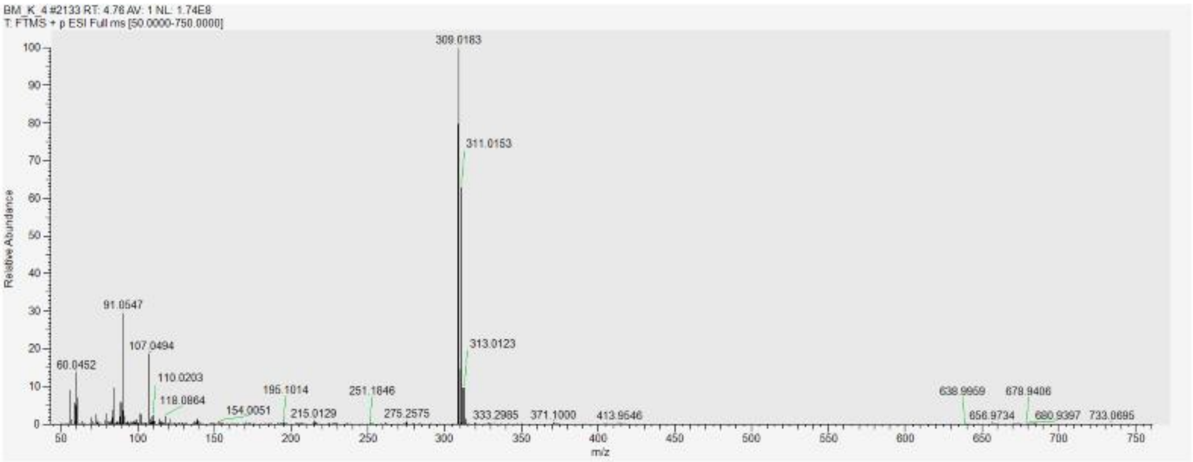

Figure S13. HR ESI–MS spectra of the compound **K4**.

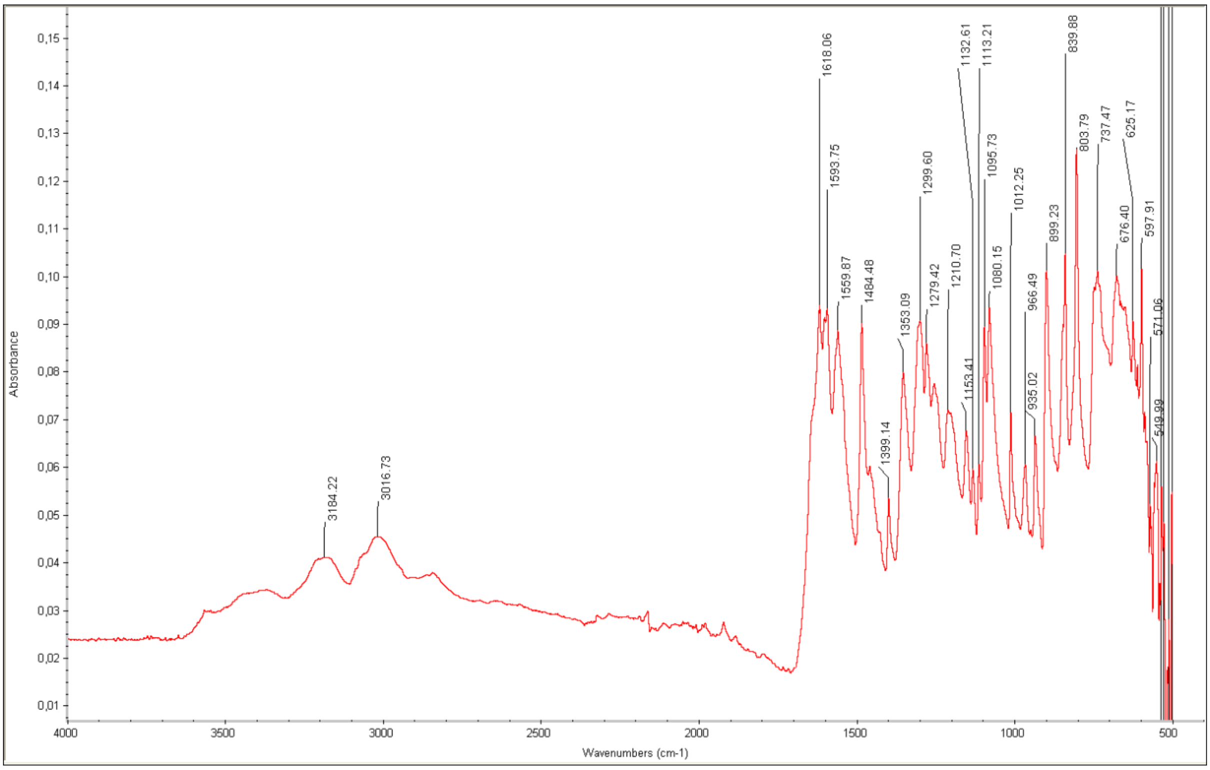

Figure S14. IR spectra of the compound **K4**.

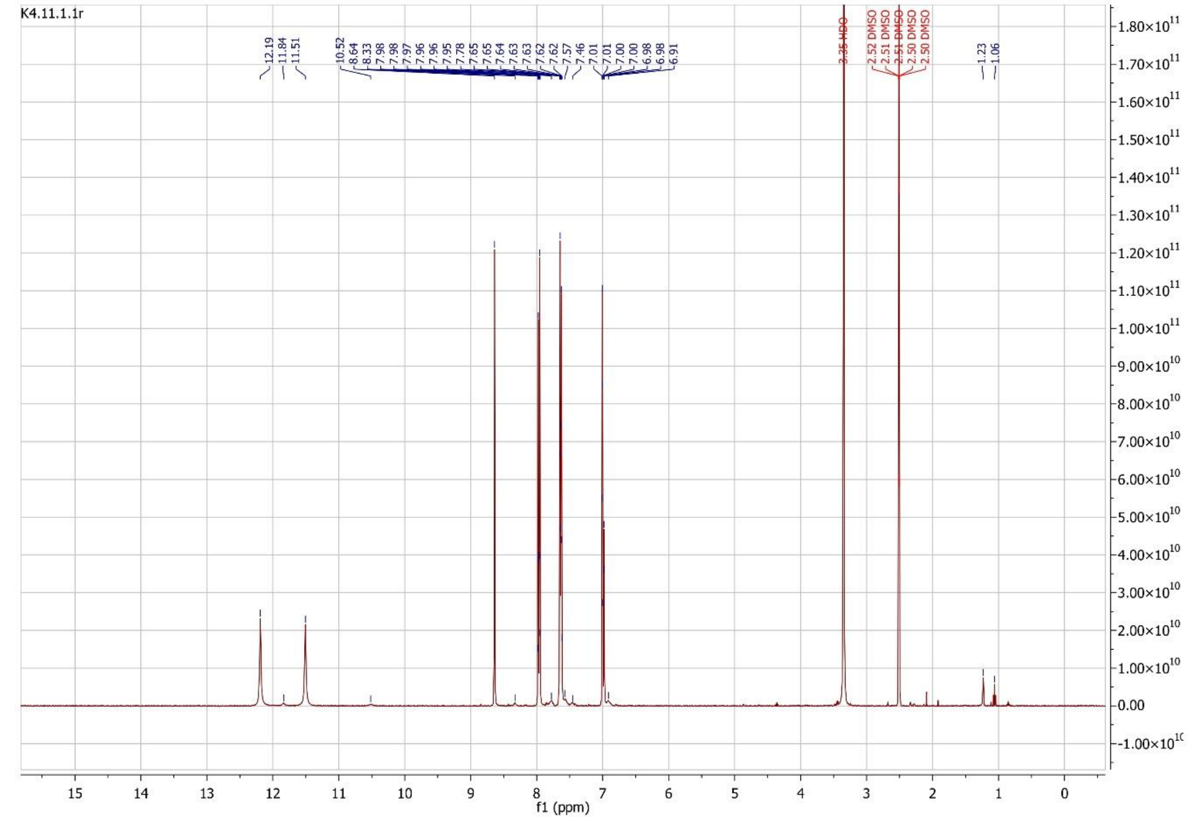

Figure S15. ^{1}H NMR spectra of the compound **K4**.

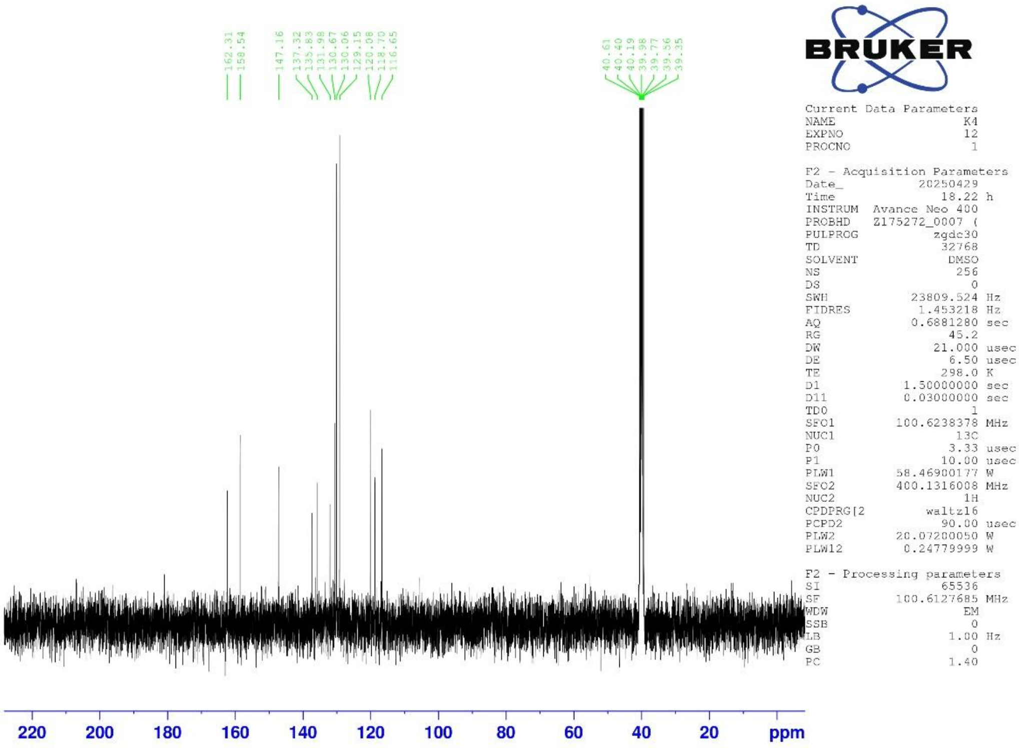

Figure S16. ^{13}C NMR spectra of the compound **K4**.

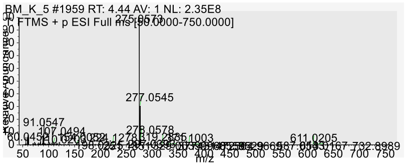

Figure S17. HR ESI–MS spectra of the compound **K5**.

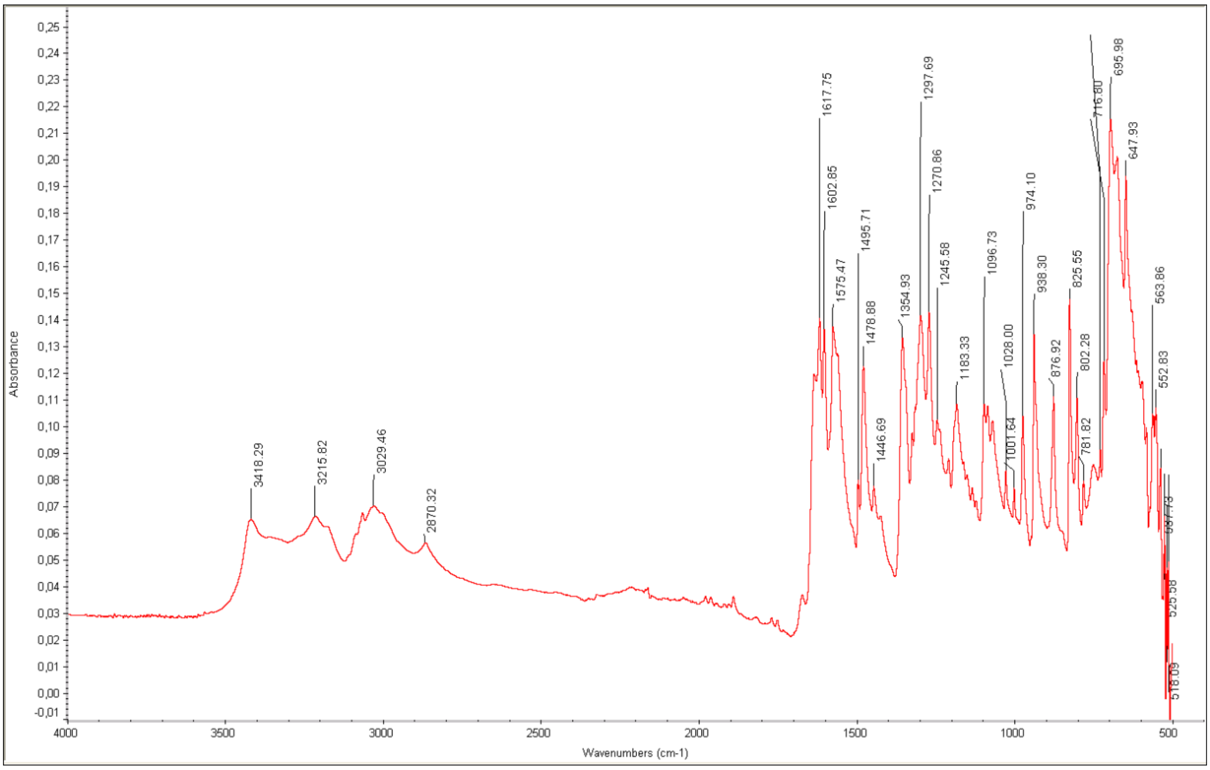

Figure S18. IR spectra of the compound **K5**.

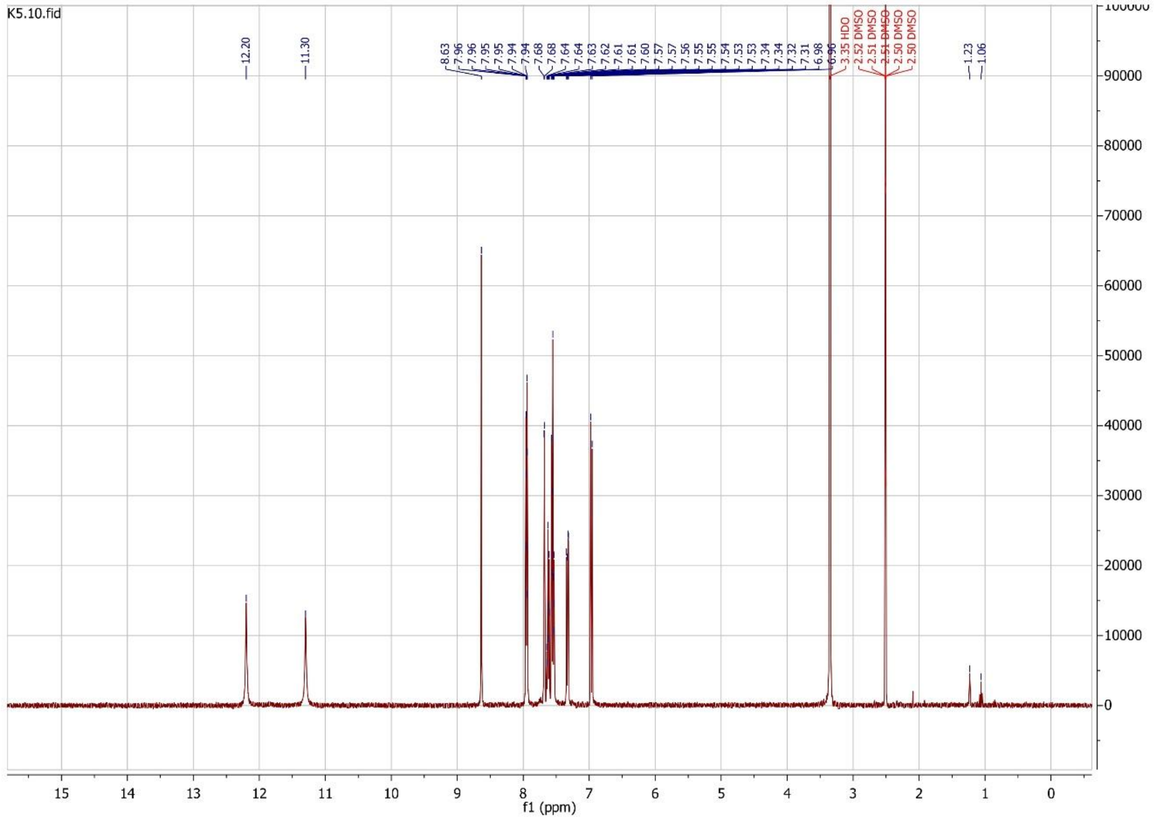

Figure S19. ^{1}H NMR spectra of the compound **K5**.

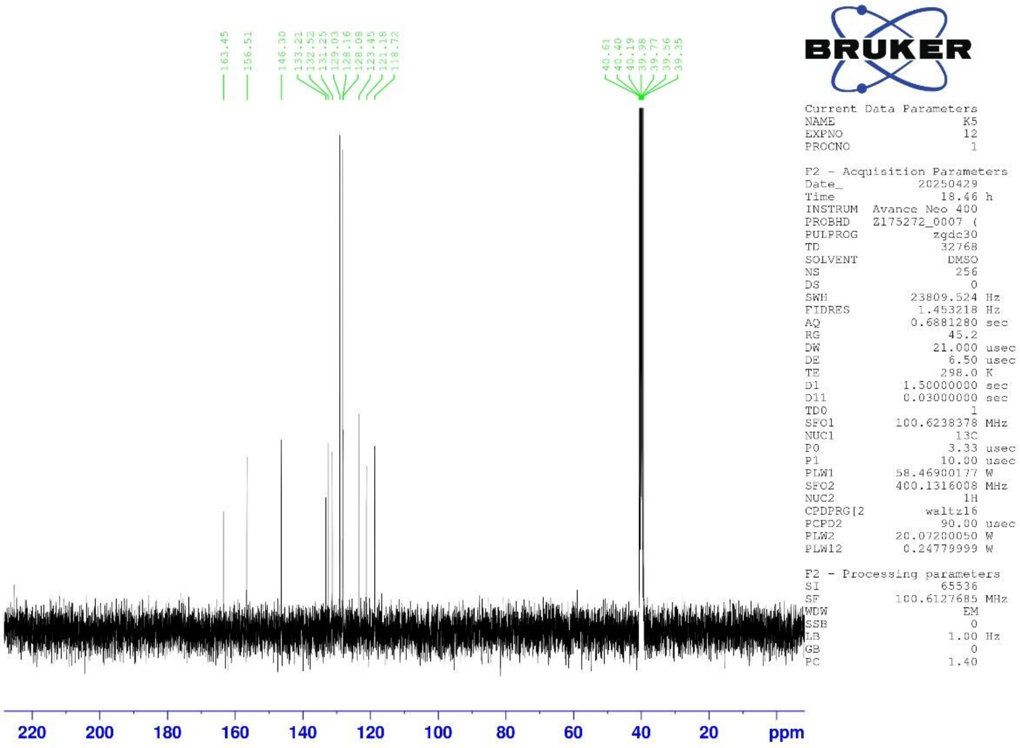

Figure S20. ^{13}C NMR spectra of the compound **K5**.

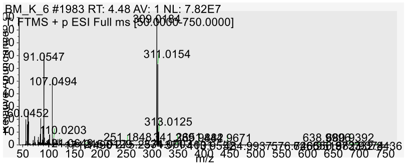

Figure S21. HR ESI–MS spectra of the compound **K6**.

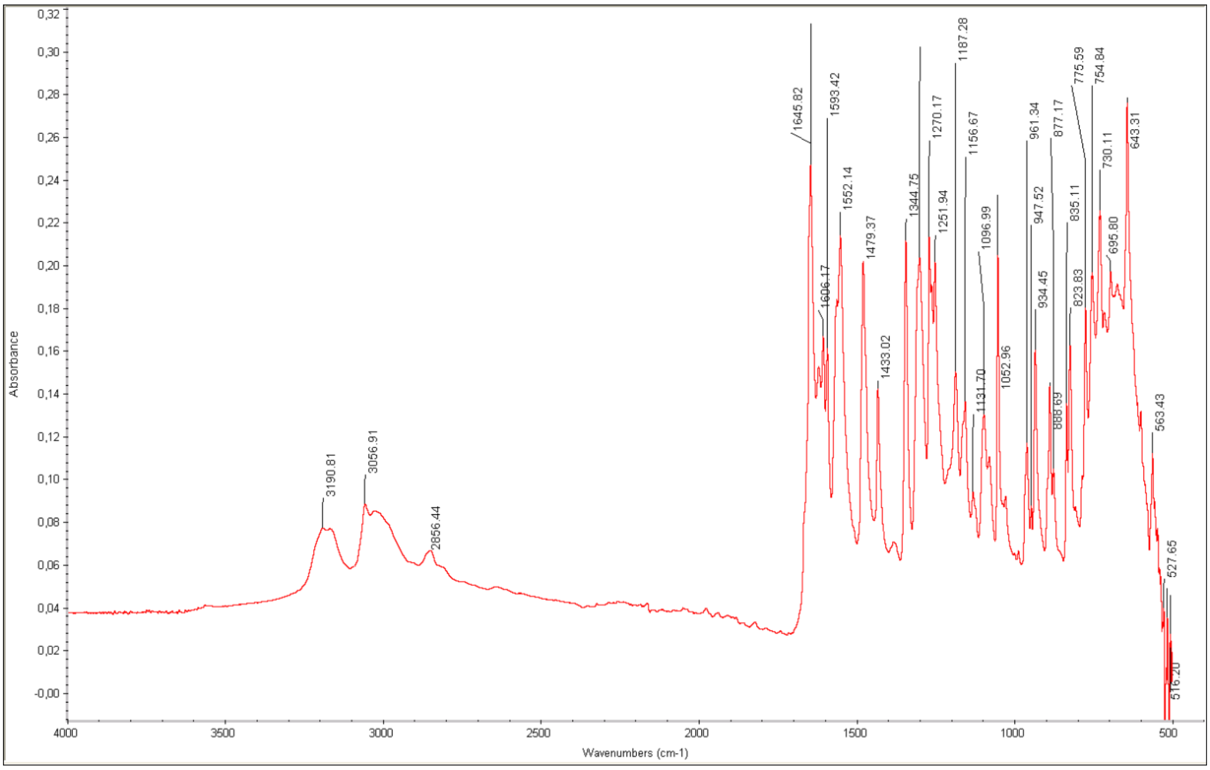

Figure S22. IR spectra of the compound **K6**.

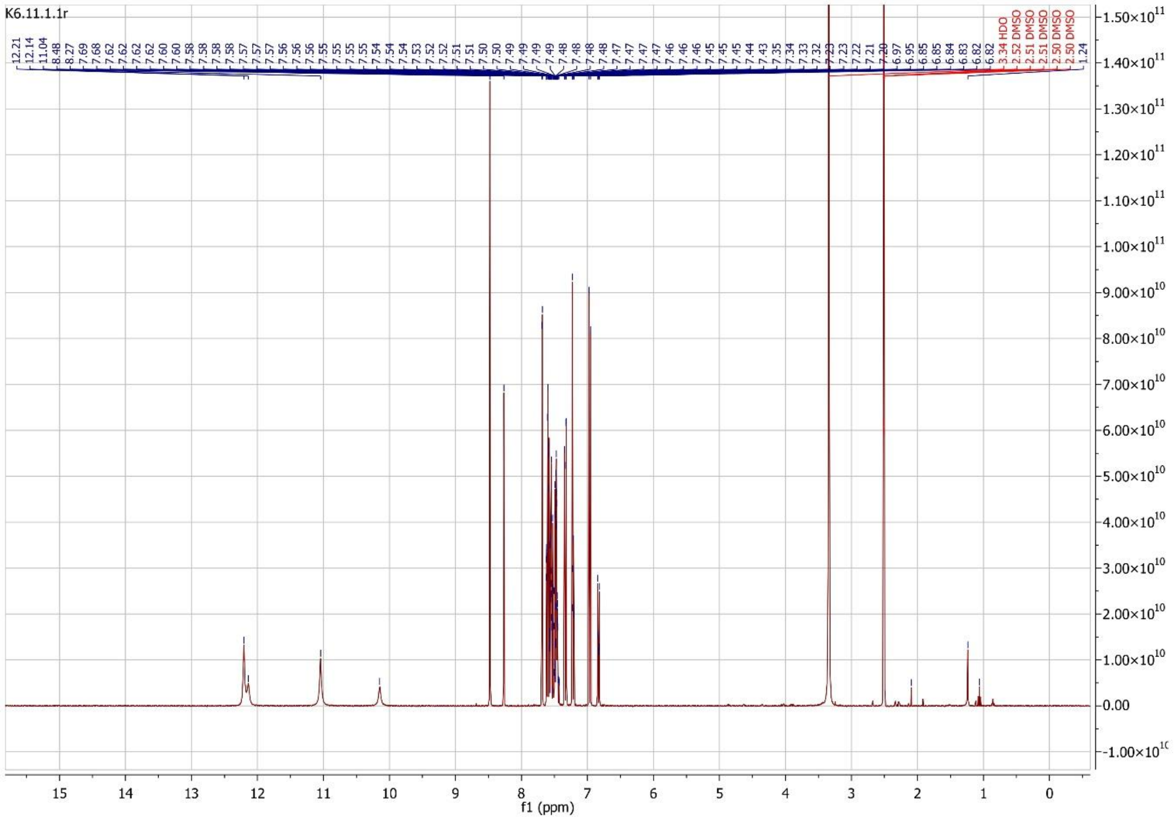

Figure S23. ^{1}H NMR spectra of the compound **K6**.

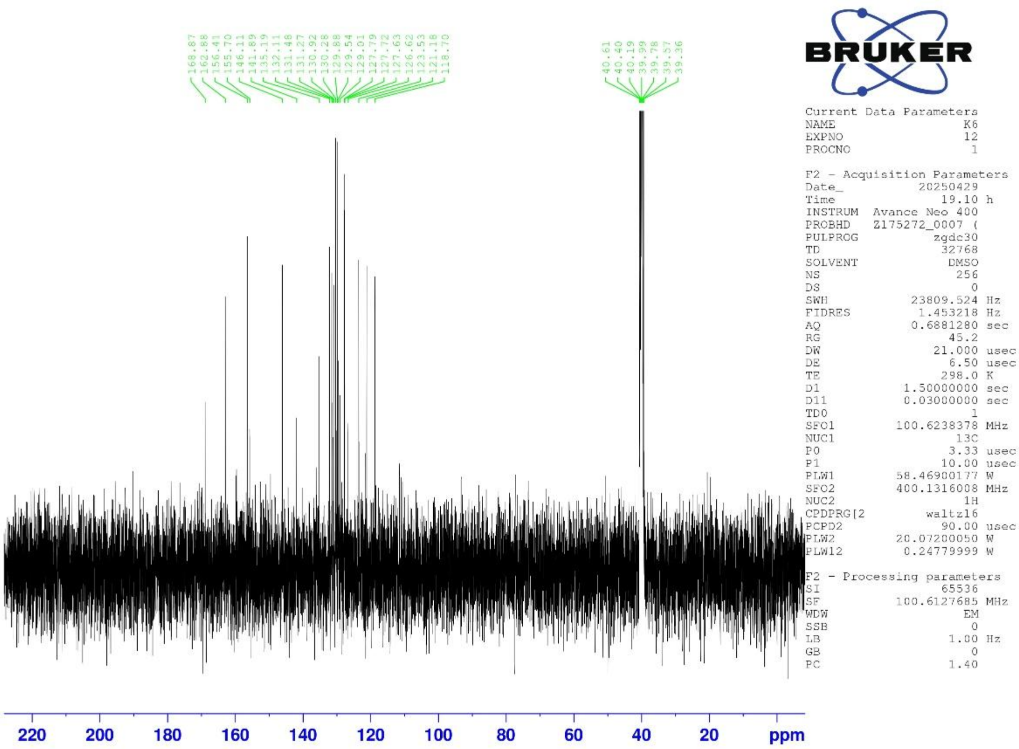

Figure S24. ^{13}C NMR spectra of the compound **K6**.

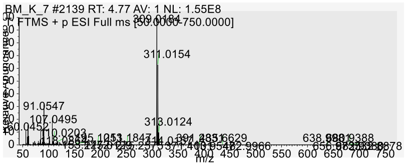

Figure S25. HR ESI–MS spectra of the compound **K7**.

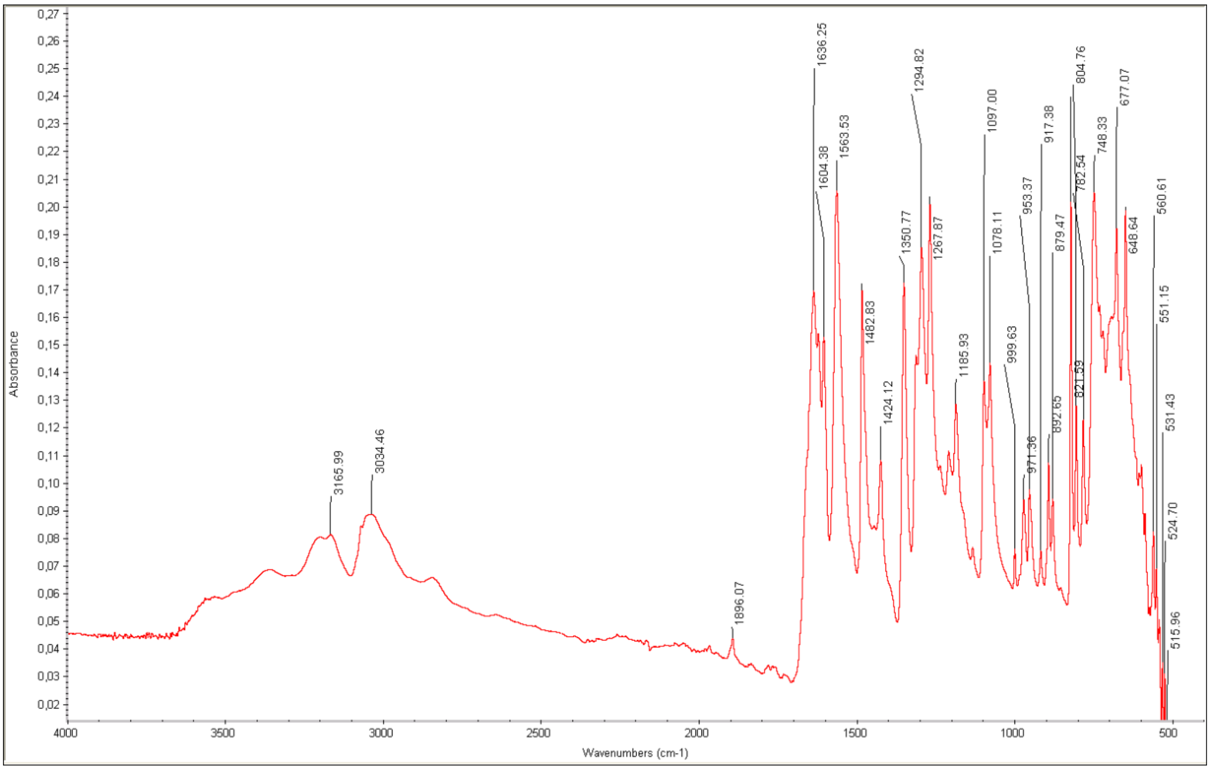

Figure S26. IR spectra of the compound **K7**.

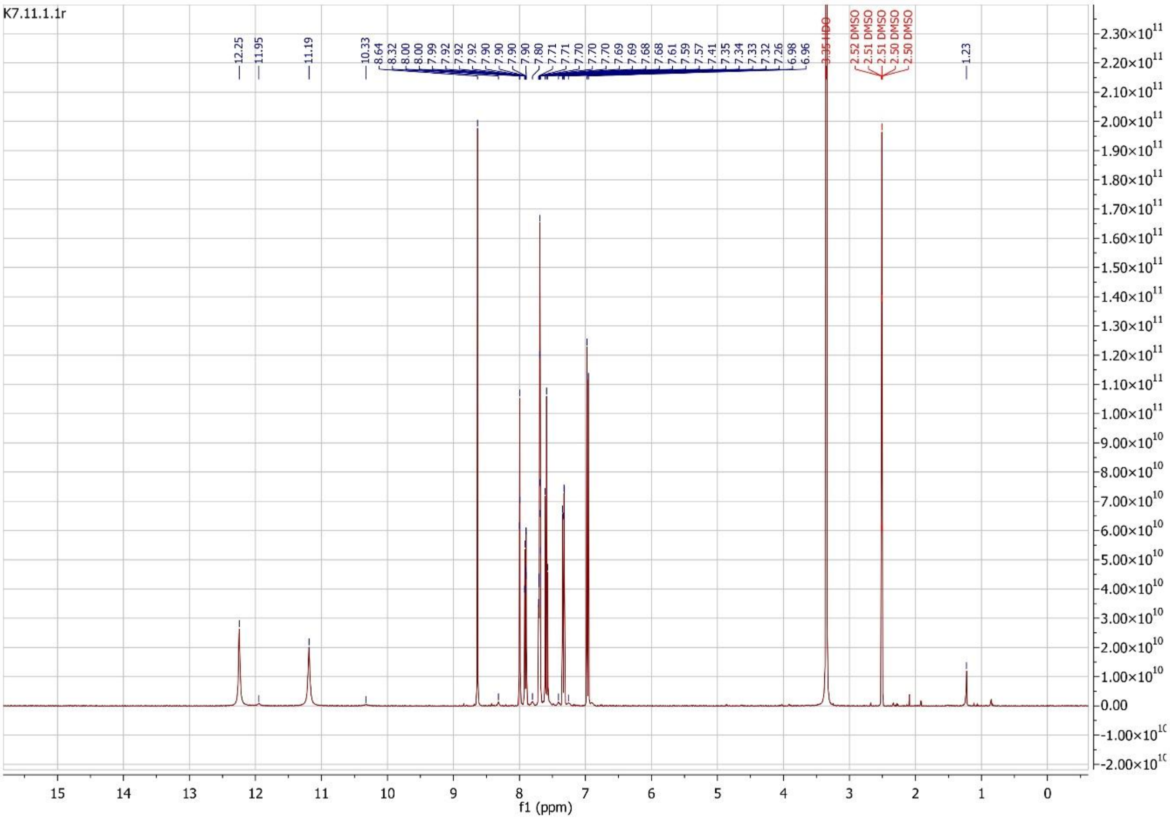

Figure S27. ^{1}H NMR spectra of the compound **K7**.

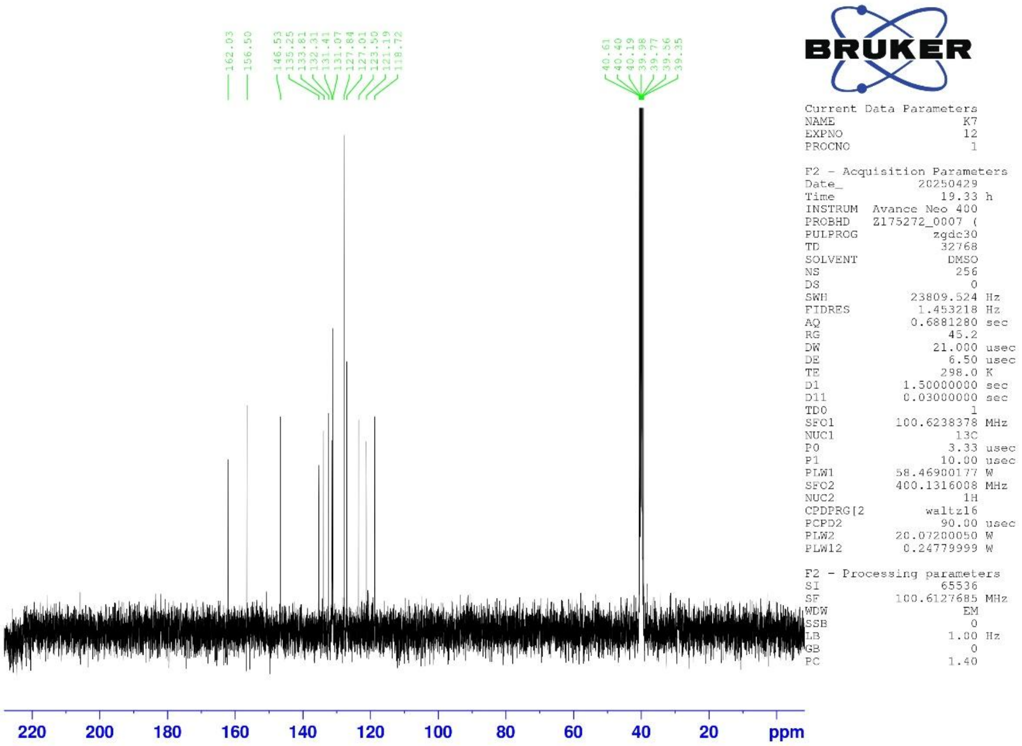

Figure S28. ^{13}C NMR spectra of the compound **K7**.

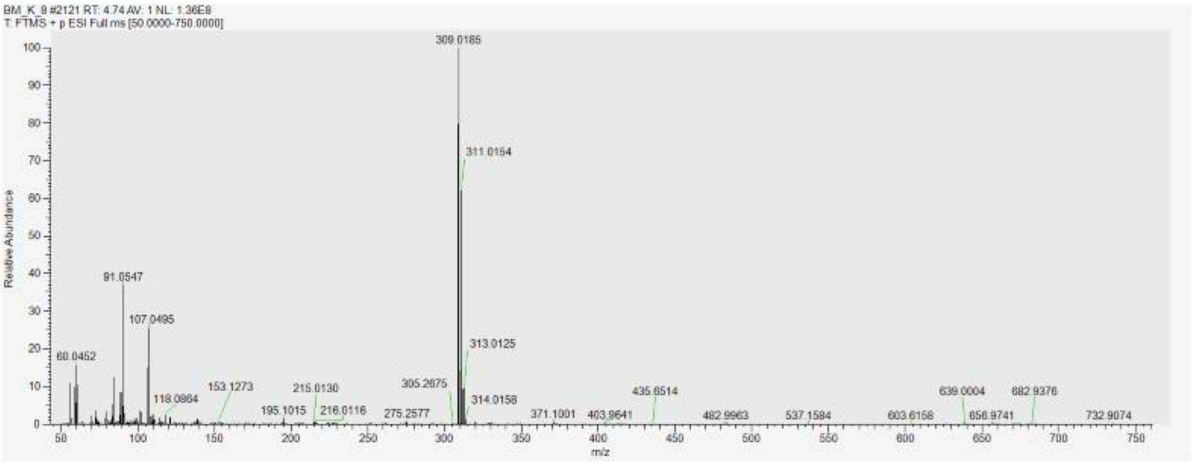

Figure S29. HR ESI–MS spectra of the compound **K8**.

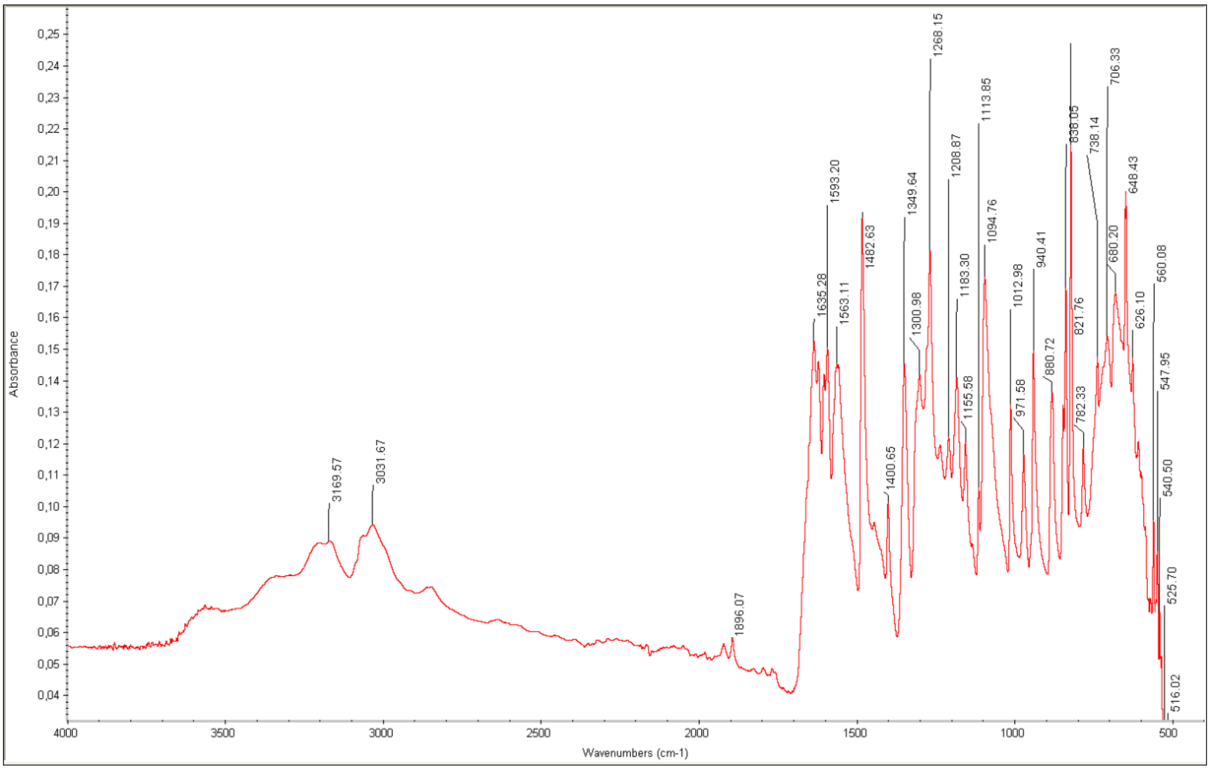

Figure S30. IR spectra of the compound **K8**.

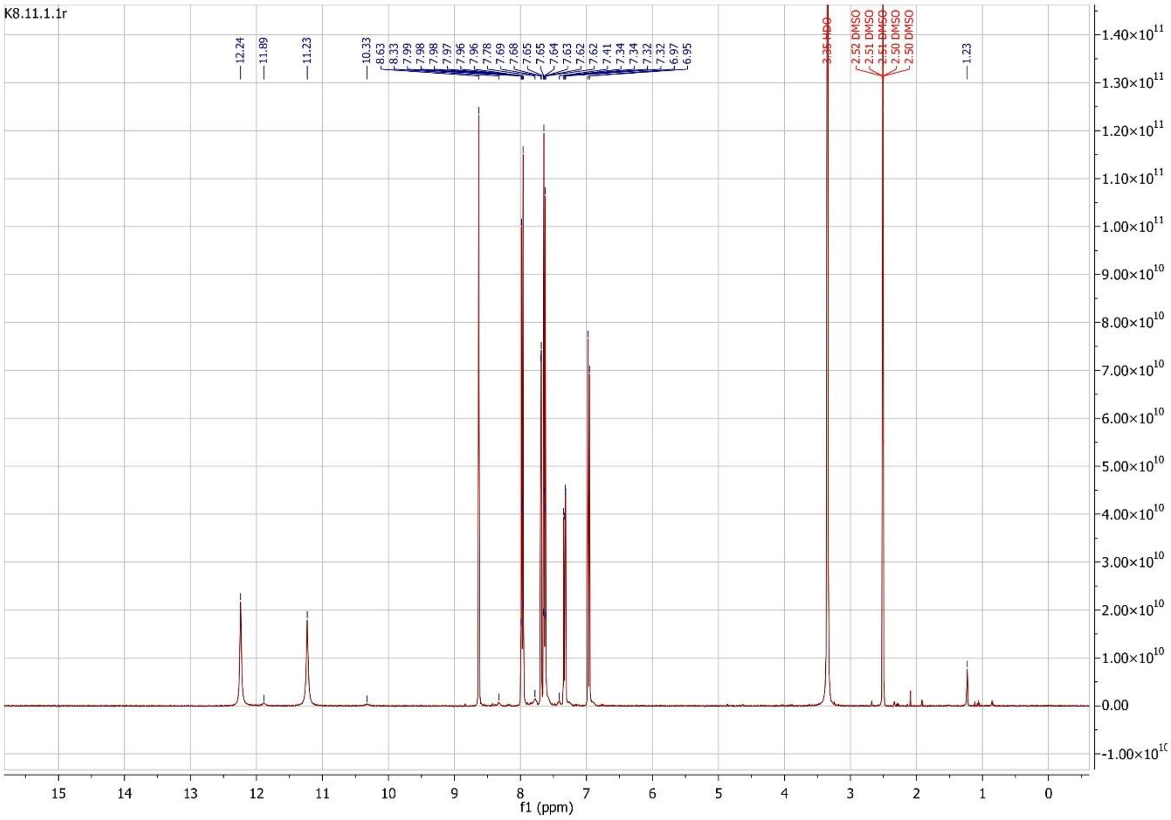

Figure S31. ^{1}H NMR spectra of the compound **K8**.

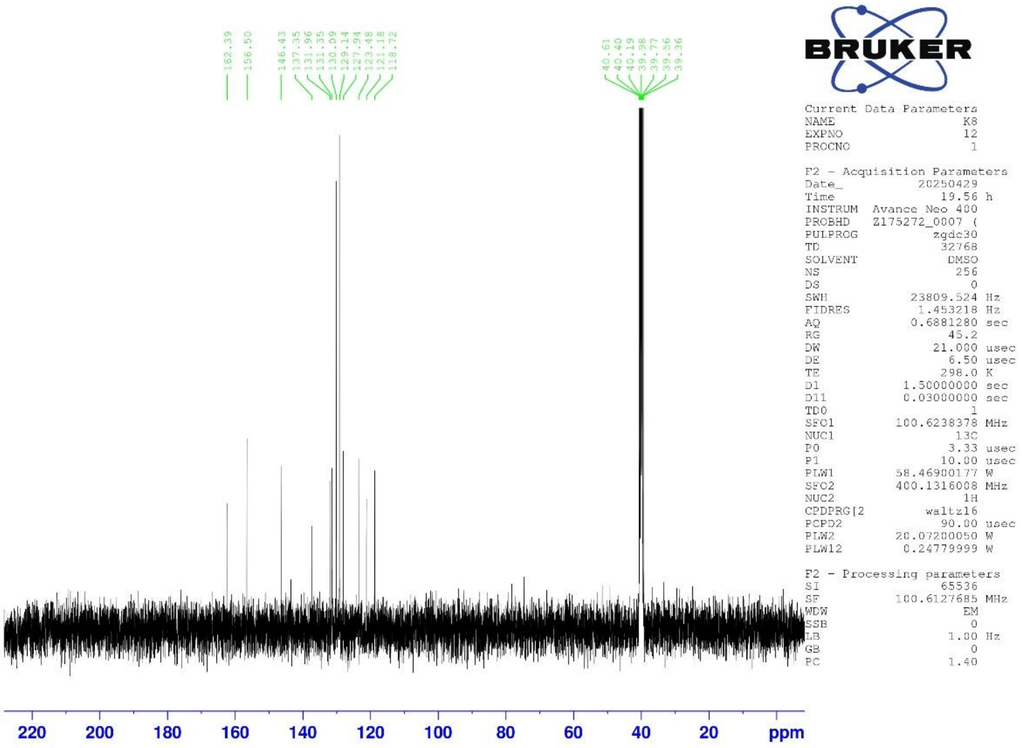

Figure S32. ^{13}C NMR spectra of the compound **K8**.
